# Assessing the Effectiveness, Engagement, and Acceptability of a Smartwatch Healthy Lifestyle Intervention for Cognitive Enhancement in Healthy Adults: Mixed Methods Electroencephalography Protocol

**DOI:** 10.2196/81490

**Published:** 2026-11-06

**Authors:** Alfredo Lopez Sherman, Juliet Fowler, Anthony Duffy, Bernhard E Riecke, Sylvain Moreno

**Affiliations:** 1 Computational Neuroscience Research Lab, School of Interactive Arts & Technology, Simon Fraser University, 13450 102 Ave #250, Surrey, BC, V3T 0A3, Canada, 1 (778) 782-9742; 2 iSpace Research Lab, School of Interactive Arts & Technology, Simon Fraser University, Surrey, BC, Canada

**Keywords:** wearable technologies, human-computer interaction (HCI), digital health, user experience (UX), cognitive function, EEG, behavioral performance, mixed methods, engagement, acceptability, healthy lifestyle intervention, brain health, adherence, smartwatch, digital health interventions, physiological data, behavior change, real-time tracking, electroencephalogram

## Abstract

**Background:**

Wearable technologies are rapidly transforming the health care landscape by enabling the tracking of real-time physiological and behavioral data used in healthy lifestyle interventions. Despite their widespread adoption in the consumer market, scientific validation of their impact on the brain remains limited, and factors influencing effects on cognition and adherence are not fully understood. This gap is critical, as consumers, health care providers, and researchers depend on these data to unlock the potential of wearables for cognitive support.

**Objective:**

We aim to assess the effectiveness, engagement, and acceptability of a commercially available smartwatch healthy lifestyle intervention on cognitive enhancement in healthy adults, using a convergent parallel mixed methods design. We seek to provide a comprehensive, replicable methodological framework to evaluate wearable technology interventions targeting cognitive enhancement in healthy adults.

**Methods:**

Healthy adults aged 18 to 40 years with a sedentary lifestyle (N=72) interested in participating in a healthy lifestyle intervention will be recruited in the Vancouver Metropolitan Area, BC, Canada. Participants will be randomly assigned into an intervention group using an Apple Watch or a control group following standard physical activity guidelines without an Apple Watch for a period of 4 weeks. Effectiveness will be evaluated through (1) cognitive function (primary outcome)—executive function, specifically inhibitory control, via electroencephalogram-based event-related potentials (N2/P3) during a go/no-go task; (2) resting-state brain activity via electroencephalogram spectral power; (3) behavioral performance (reaction time and accuracy); (4) perceived health (36-item Short Form Health Survey); and (5) qualitative analysis of factors affecting the intervention’s effectiveness. Engagement will be assessed through goal completion metrics and qualitative analysis of barriers, facilitators, and usage patterns. Acceptability will be assessed through System Usability Scale scores and qualitative analysis of factors affecting usability, satisfaction, and user preferences. This design integrates assessments before, during, and after intervention. Qualitative assessments will include weekly check-ins and semistructured interviews to obtain a comprehensive understanding of factors influencing the effectiveness, engagement, and acceptability of the intervention. Exploratory outcomes include descriptions of possible interactions between the 3 constructs and comparison of smartwatch-tracked vs self-reported goal completion.

**Results:**

The study received funding from the AGE-WELL Network Centres of Excellence (April 2020-March 2023) and was approved by the Simon Fraser University Research Ethics Board. Enrollment for the study is ongoing. As of October 2026, data have been collected from 48 participants and are currently being processed and analyzed. The first results are expected to be published in 2027.

**Conclusions:**

This study outlines a foundational framework for evaluating the effectiveness, engagement, and acceptability of wearable health interventions targeting cognitive enhancement in healthy adults. Furthermore, our mixed methods approach with randomized controlled comparison is expected to inform the design and development of effective, engaging, and friendly wearable brain health technologies.

## Introduction

Every year, hundreds of commercial brands develop new wearable devices for the consumer market [1]. The global wearable technology market had an estimated worth of US $40.65 billion in 2020 and is projected to triple in value by 2028 (US $118.16 billion) [2]. Advances in wearable sensors over the last decade have enabled the gathering of real-time physiological and behavioral data in an increasingly precise and unobtrusive way [3]. The sensors embedded in wearables gather movement, physiological, and biochemical data for a variety of tasks such as tracking steps, measuring heart rate, and monitoring glucose, respectively [4]. These data provide a wealth of information on people’s behaviors, emotions, and lifestyles, ultimately providing users with personalized health data for health monitoring and behavior change [5]. It also allows researchers and clinicians to explore and obtain keener insights into brain health [3,6] and could be key toward designing effective multimodal personalized interventions using technologies already familiar to users [7]. Technological advances in wearable sensors may also reduce the gap between medical and nonmedical grade devices [8], advancing digital interventions to better support brain health [2,7] and potentially extending its range to hard-to-reach populations such as rural and low-resource locations [3,4]. Research in wearables holds the potential to revolutionize the next generation of brain health interventions [6].

Wearable devices have changed the health technology industry by empowering users to become active and responsible drivers of their own care, but these benefits will only materialize if people continually use and adopt these products [9,10]. Benefits such as early health issue prediction will only be realized through the continuous collection and analysis of comprehensive sets of data, which could also be used to identify and promote positive lifestyle habits [10]. However, designing a wearable associated with a healthy lifestyle and providing scientific evidence to support this assumption while keeping up with the rapid nature of the field is often challenging [5,11]. Studies report different outcomes of continual use of wearables over long periods of time [5,11], with a key study providing evidence that 32% of users abandon wearables after 6 months of use [5]. Continual use of wearables is impacted by user experience conditions such as comfort [12], effort [7], gamification [11], confidence [5,6], and social observability [9], as well as the type of goal pursued by the user. Some researchers have categorized these goals as intrinsic and extrinsic. Intrinsic goals are satisfying to pursue and include the innate psychological needs for autonomy, relatedness, competence, and growth. Extrinsic goals are focused on obtaining rewards and positive evaluations from others, sometimes leading to stressful behavior with unsatisfactory outcomes [9,13]. Exploring user experience factors that influence adherence and long-term efficacy, including the role of intrinsic and extrinsic goals in outcomes, is essential toward the increased viability of wearable-based brain health interventions [14].

While there are some positive indications that using wearable trackers improves overall health [1] and evidence that comprehensive lifestyle interventions could enhance cognition [15], evidence of the effect of wearables on the brain still needs to be established with scientific validation. It is common to find unsubstantiated scientific claims on company websites and marketing materials, which could set a dangerous precedent if misinterpreted [4]. Similarly, single-subject, subjective reports available from Quantified Self movements are scientifically unreliable. Despite extensive research focusing on wearables for disease monitoring and clinical populations, it remains unclear how healthy adults regularly exposed to a constant stream of behavioral and physiological feedback from wearables experience cognitive enhancement effects [5]. Furthermore, the complex, rapidly evolving nature of wearable technologies presents challenges in evaluating their impact. For example, users’ context and lifestyle could produce different outcomes in various scenarios [16]. These are important factors that must be explored in the scientific community.

With the goal of evaluating whether wearables can enhance cognitive function in healthy adults, we propose a study to assess the effectiveness, engagement, and acceptability of a commercially available smartwatch healthy lifestyle intervention on executive function enhancement, specifically inhibitory control. Executive functions are foundational processes for higher-order cognition, focus, and behavioral regulation [17], and evidence suggests that they can be enhanced through lifestyle interventions [15].

To evaluate comprehensively the effect of a smartwatch healthy lifestyle intervention on cognitive function, we will assess 3 essential, interdependent constructs. Effectiveness will measure whether the intervention produces the expected cognitive, behavioral, and perceived health outcomes, with executive function (inhibitory control) as the primary outcome. Engagement will assess whether participants actively interact and commit to the intervention as expected, an essential prerequisite for effectiveness to occur. Acceptability will evaluate whether participants find the intervention usable and satisfactory, another essential precondition for effectiveness and engagement to occur. The proposed 3-construct framework follows established guidelines for evaluating complex health behavior change technologies in human-computer interaction (HCI) research [18,19].

Our objectives are as follows:

1. to assess the effectiveness of a smartwatch healthy lifestyle intervention on cognitive enhancement in healthy adults, with inhibitory control as the primary outcome, along with secondary effectiveness outcomes including resting-state brain activity, behavioral performance, and perceived health;
2. to evaluate the effectiveness, engagement, and acceptability of the intervention through a convergent parallel mixed methods approach combining quantitative and qualitative assessments; and
3. to provide a comprehensive, replicable methodological framework for evaluating wearable technology interventions targeting cognitive enhancement in healthy adults.

Our research methodology was inspired by previous work in the field, specifically the studies conducted by Moreno et al [20], Antle et al [21], Bevan Jones et al [22], and Kiaei Ziabari et al [23]. To refine our protocol, we conducted pilot testing from May 2023 to September 2023 with volunteers (N=4). Data from this pilot are not reported, as procedures evolved and were not standardized for all volunteers. The current protocol incorporates these refinements.

## Methods

### Study Design and Population

#### Recruitment and Consent

Seventy-two healthy adults (control: n=36, intervention: n=36) will be recruited in the metropolitan area of Vancouver, BC, Canada. Sample size was determined through a power analysis for our primary cognitive outcome. We conducted an *F*-test power analysis for a mixed repeated measures design examining the effect of group (intervention vs control), time (pretest vs posttest), and condition (go vs no-go trial) on event-related potential (ERP) amplitude. Based on the effect sizes of comparable lifestyle interventions (ηp²≈0.2) [20], our analysis yielded 98% power to detect ηp²=0.2 with N=72 and α=.05. This sample size also provides adequate power for our other outcomes and will be above typical samples for mixed methods HCI research [24] and ERP studies [25].

A recruitment poster with study details, contact information, and a link to the prescreening survey will be distributed both physically and digitally for its use in physical channels such as local bulletin boards and displays. Study personnel will also contact prospective participants through snowball recruitment, the mailing and recruitment lists of local universities (Simon Fraser University and University of British Columbia), newsletters, and known contacts. Initial contact will be made by email. Individual contact with prospects will be made by email and phone call to schedule in-person research activities.

#### Ethical Considerations

This study was reviewed and approved by the Research Ethics Board of Simon Fraser University (study 30001509). The study will be conducted following the Research Ethics Board regulations. All participants will provide written informed consent before any assessment. Participants and prospects will be instructed to not consume alcohol or drugs 24 hours before assessments. They should also have dry hair and avoid using hair products and makeup, as they may interfere with electroencephalogram (EEG) recording procedures [25].

#### Inclusion Criteria

We will recruit healthy adults aged 18 to 40 years interested in improving their lifestyle to participate in our intervention. Participants must have a sedentary lifestyle indicated by self-report of 1 day or less of weekly moderate exercise. They must have normal or corrected-to-normal vision and no color vision deficiency. They must own and actively use an iPhone XS or later with iOS 17 or later, due to compatibility restrictions with the smartwatch used for the intervention. Apple Watch was selected due to its widespread adoption [26]. This provides a more comprehensive representation of the current impact of smartwatch healthy lifestyle interventions on cognitive function. The participants must also be available to attend all remote and in-person study sessions required for the intervention in a designated lab space. Lastly, participants must be right-handed due to differences in the laterality of brain activity [25].

#### Exclusion Criteria

We will exclude prospects who report significant health problems, currently participate in a health intervention, currently use a smartwatch with healthy lifestyle features, or have used one in the past year. If applicable, prospects will be asked to describe their current physical activity habits in detail, including type, frequency, duration, and intensity. Prospects who report engaging in moderate exercise (eg, running and cycling) for 2 or more days weekly will be excluded. Prospects will undergo the first pretest assessments for a final screening. Participants with an IQ score of grade IV or lower (the lower 25 percentile group, determined using the Raven Standard Progressive Matrices [RSPM] scale) will be excluded. We will request prospects and participants to report medication use and will exclude them if these could affect any of the assessments.

#### Randomization

To reduce pre-existing differences between groups, participants will be assigned using covariate-adaptive pseudorandomization, a procedure recommended for clinical and experimental trials with continuous enrollment, where baseline variables are known to influence primary outcomes and where true random allocation risks producing imbalanced groups [27]. This approach has been previously used in related published work [20,28,29]. To prevent predictability, the first 10 participants will be assigned by true random allocation before covariate-adaptive procedures are applied. For subsequent participants, temporary assignment to both groups will be evaluated using *t* tests for each covariate. The group with the higher minimum *P* value, indicating better overall balance, will be selected for final assignment. See Figure 1 for more details.

Covariates controlled between groups (*t* test: *P*>.05) will include age; sex assigned at birth; and preintervention measurements of IQ (RSPM), perception of their own health (Short Form Health Survey [SF-36]) [30], behavioral performance (reaction time and accuracy), and cognitive measurements (ERPs).

**Figure 1.**
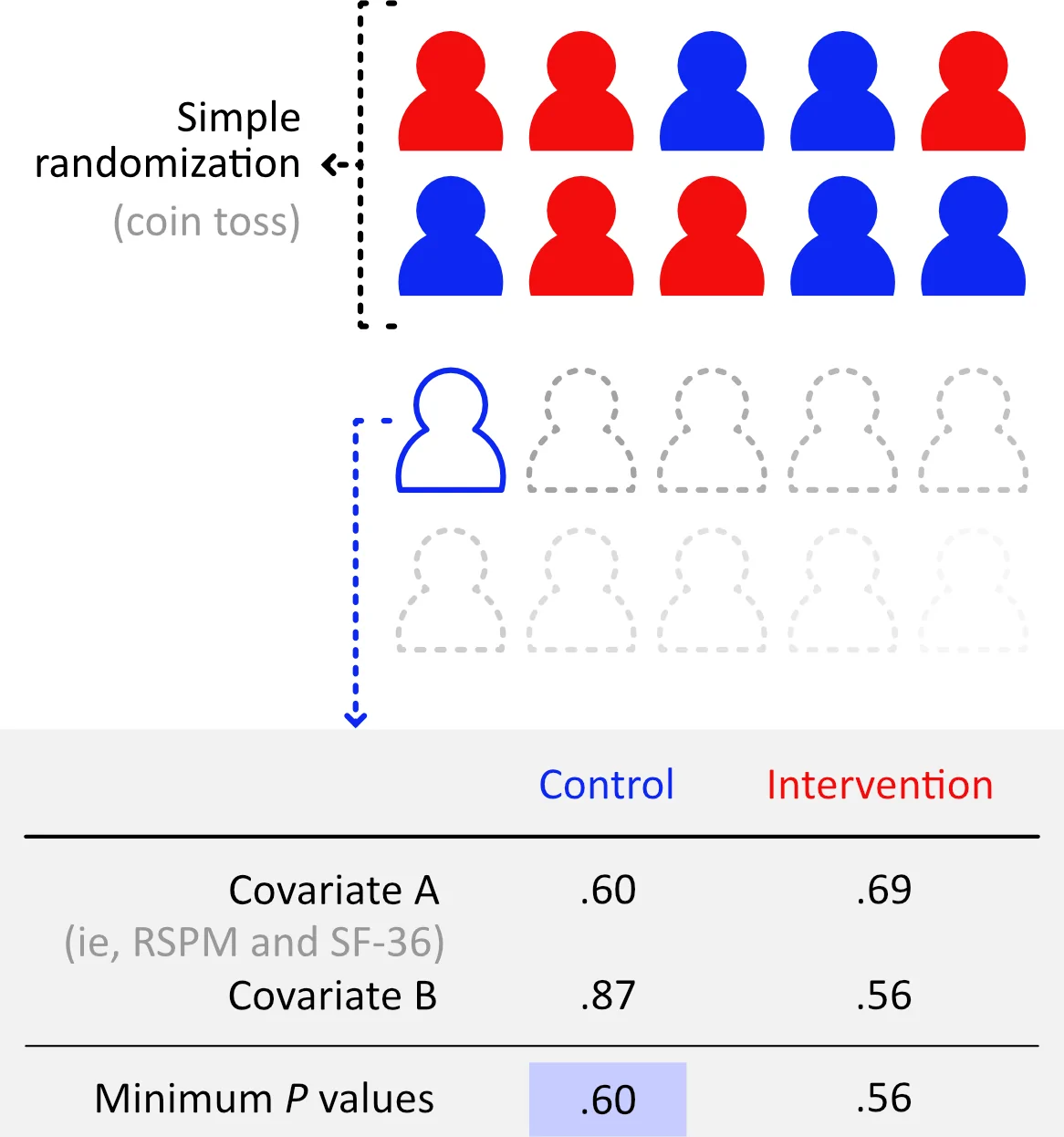
Example breakdown of the first 11 participants’ group assignment. In this case, the 11th participant will be assigned to the control group because of the higher minimum *P* value (.60>.56). RSPM: Raven Standard Progressive Matrices; SF-36: Short Form Health Survey.

#### Procedure

The study will consist of 4 phases: pretest, onboarding, intervention, and posttest. All phases are scripted (Multimedia Appendix 1) and will be led by a team of 2 research assistants. Figure 2 illustrates an overview of the study procedure.

**Figure 2.**
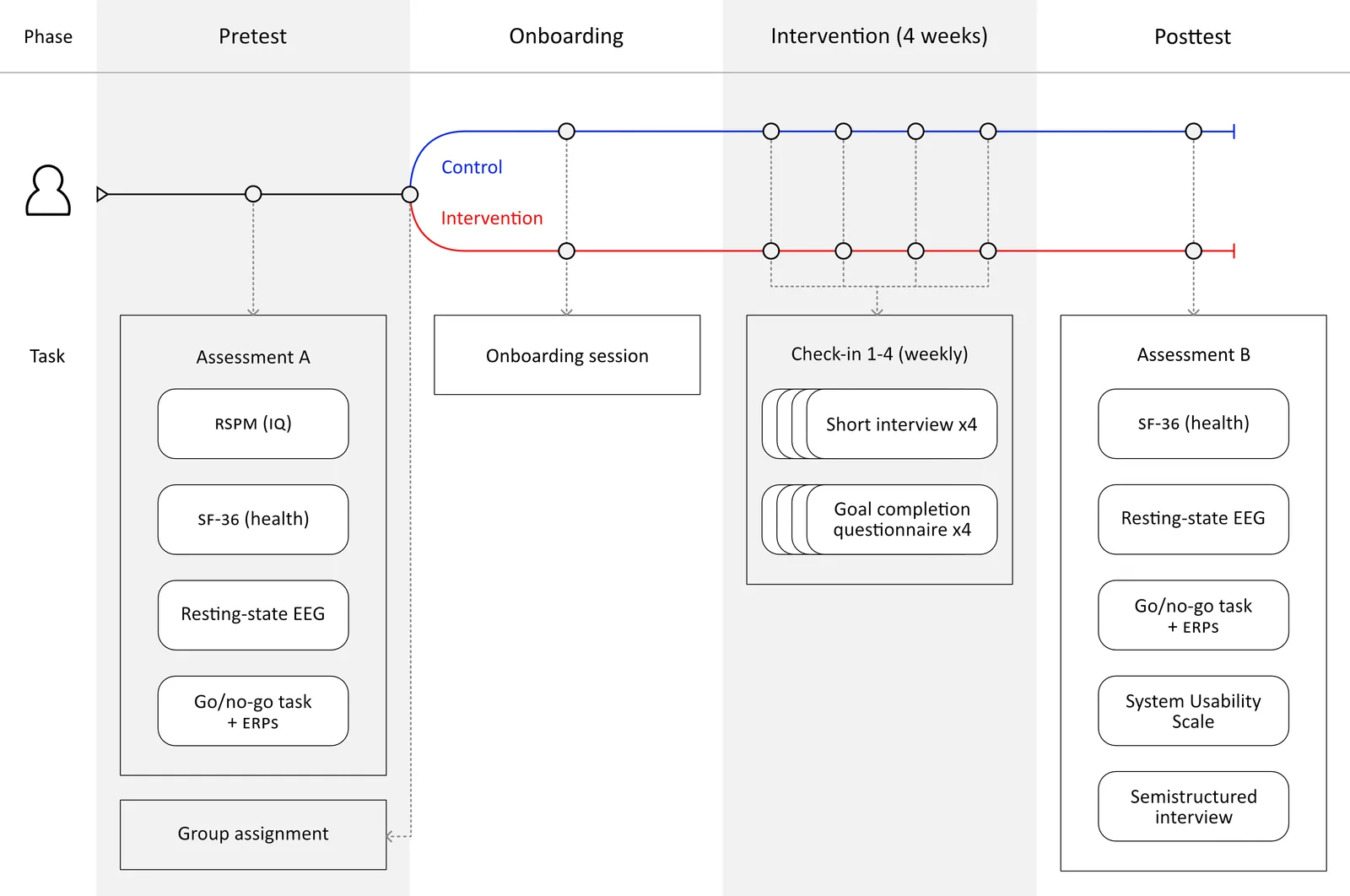
Overview of the study protocol, including all 4 phases (pretest, onboarding, intervention, and posttest), tasks, and assessments considered for each task. Outcome measures used for group assignment in addition to age and sex assigned at birth included RSPM (IQ), SF-36 (health) and go/no-go task + ERPs. EEG: electroencephalogram; ERP: event-related potential; RSPM: Ravens Standard Progressive Matrices; SF-36: Short Form Health Survey.

##### Pretest

This session will last approximately 60 to 90 minutes. After signing the consent form and being assigned a participant ID, the pretest will begin. Participants will begin by answering an IQ assessment (RSPM) on the computer (Multimedia Appendix 1). In this IQ assessment, participants will be shown a pattern with a piece cut out. They must think about the piece needed to complete the pattern correctly, choosing the right piece from 6 options. The research team will guide participants through a sample exercise and answer any questions before starting the IQ assessment. There will be no time limit for the test, but participants will be encouraged to finish as fast as they can. We will record their completion time. Finally, we will exclude participants who do not satisfy the IQ assessment criteria. RSPM was selected as an IQ assessment for 2 reasons: as a brief, nonverbal screening tool to ensure that participants meet the minimum cognitive criteria for study participation (above the bottom 25 percentile group) and as a baseline covariate in our randomization procedure to control for individual differences in general cognitive ability between groups.

Selected participants will then be guided through the EEG setup. We will use a BioSemi Active Two system for the EEG assessments. The BioSemi allows real-time visualization of the electrical signal of the brain during tasks and recordings. A cap with 64 electrodes will be used, in addition to common mode sense (CMS) and driven right leg (DRL) electrodes, as well as 5 external electrodes to track eye and muscle movement (2 electrooculogram [EOG] electrodes on the temples, 1 EOG electrode beneath the left eye, and 2 electrodes on the mastoids). The skin area where the external electrodes are to be placed will be cleaned using alcohol swabs, and the head will be measured (from the nasion to the inion) to select a well-fitted cap. After conductive gel is applied to the external electrodes to improve signal and the electrodes are secured in place with electrode collars, the cap will be placed on the participant’s head, tested for correct placement and fit, and secured using a chin strap. Then, conductive gel will be applied to each of the 64 electrode locations using a plastic syringe with circular motions to get the hair out of the way and improve the connection between the electrode and the scalp, followed by the insertion of the pin-type electrodes. Finally, the connection of each electrode will be tested and adjusted for connection flaws (impedance ±25 mV and visual inspection of noisy signal) on a separate EEG acquisition computer before the recording.

While the EEG system is being set up by the research team, participants will be asked to answer the SF-36 (RAND Corporation; Multimedia Appendix 2) on the computer. The SF-36 captures the self-perception of health and well-being. The survey has 36 items grouped into 8 dimensions, including physical functioning, physical and emotional limitations, vitality, social functioning, bodily pain, general health, and mental health. It also includes a health change dimension evaluating the perceived improvement of health after a period of time. The SF-36 is a widely validated survey with strong reliability estimates (>.80) and sensitivity to changes over time [30,31]. We will administer the complete SF-36 to capture potential broader health benefits of the intervention and to facilitate comparison with other intervention studies. The research team will state that the survey is not a test, inviting participants to choose the response that best represents how they currently feel, remaining available for any questions while the participant completes the survey.

After completing the survey and finishing setting up the EEG system, we will record the participant’s brain activity at rest. Instructions will be provided both verbally and visually on the computer screen (Figure 3). Participants will be asked to focus on a fixation cross for 1 minute and to avoid moving or blinking. The research team will ensure communication between the stimulus and recording computers and then begin the recording. The participant will initiate the 1-minute task when they are ready by clicking on the screen. After completing this task, instructions for a second 1-minute task will be provided. This time, participants will close their eyes and avoid moving while not focusing on anything in particular. The participant will click on the screen when they are ready. The research team will let them know when the task is over and stop and save the recording on the EEG system.

**Figure 3.**
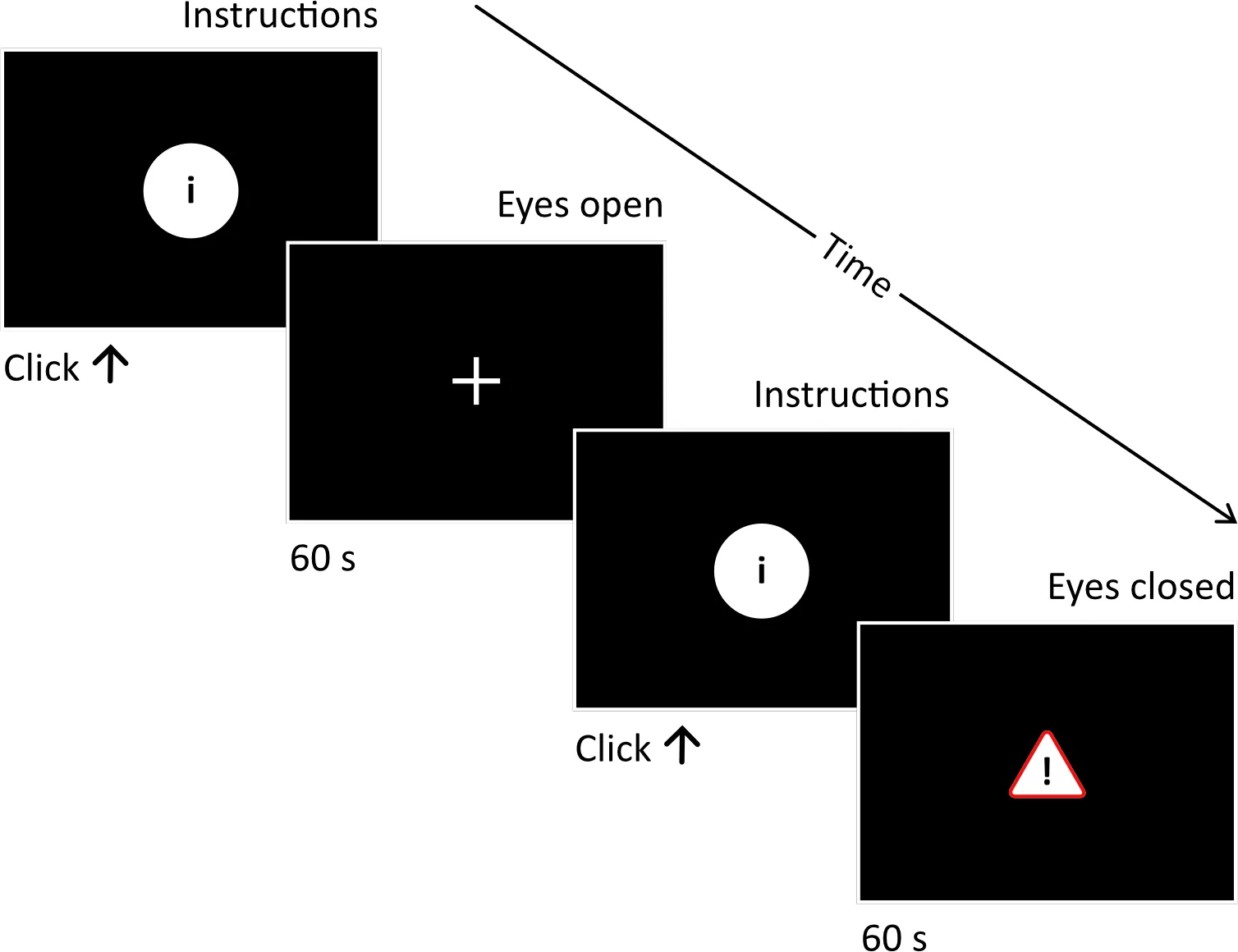
Schematic of the sequence of prompts for the resting-state task.

Next, participants will perform a go/no-go task while the EEG is being recorded. The go/no-go task is used to assess the efficiency of response inhibition. Specifically, it measures the capacity to control responses and sustain attention [20]. The task will consist of 2 stimuli differing in color and shape, for which the participants will either respond (clicking a mouse button) or withhold a response (not clicking a mouse button), depending on whether a go or no-go stimulus is displayed on the screen (Figure 4). The task will consist of 160 trials. Of these, 80% (n=128) will present the go stimulus, while the rest (20%, n=32) will present the no-go stimulus. The order of trials is randomized for each go/no-go task, decreasing the chances of predicting the no-go stimuli. The roles of the stimuli will be randomized for each participant. After a task is defined and the signal is tested, instructions will be provided both verbally and visually on the computer screen. Participants will be asked to respond as fast and as accurately as they can while they avoid moving or blinking. They will do a short practice task (10 trials) to ensure comprehension of the instructions. After the practice is complete, the research team will start the recording and ask the participant to click on the screen when they are ready to begin the main task. When the go/no-go task is completed, the research team will remove the EEG electrodes and cap and explain the next steps of the intervention to the participant.

**Figure 4.**
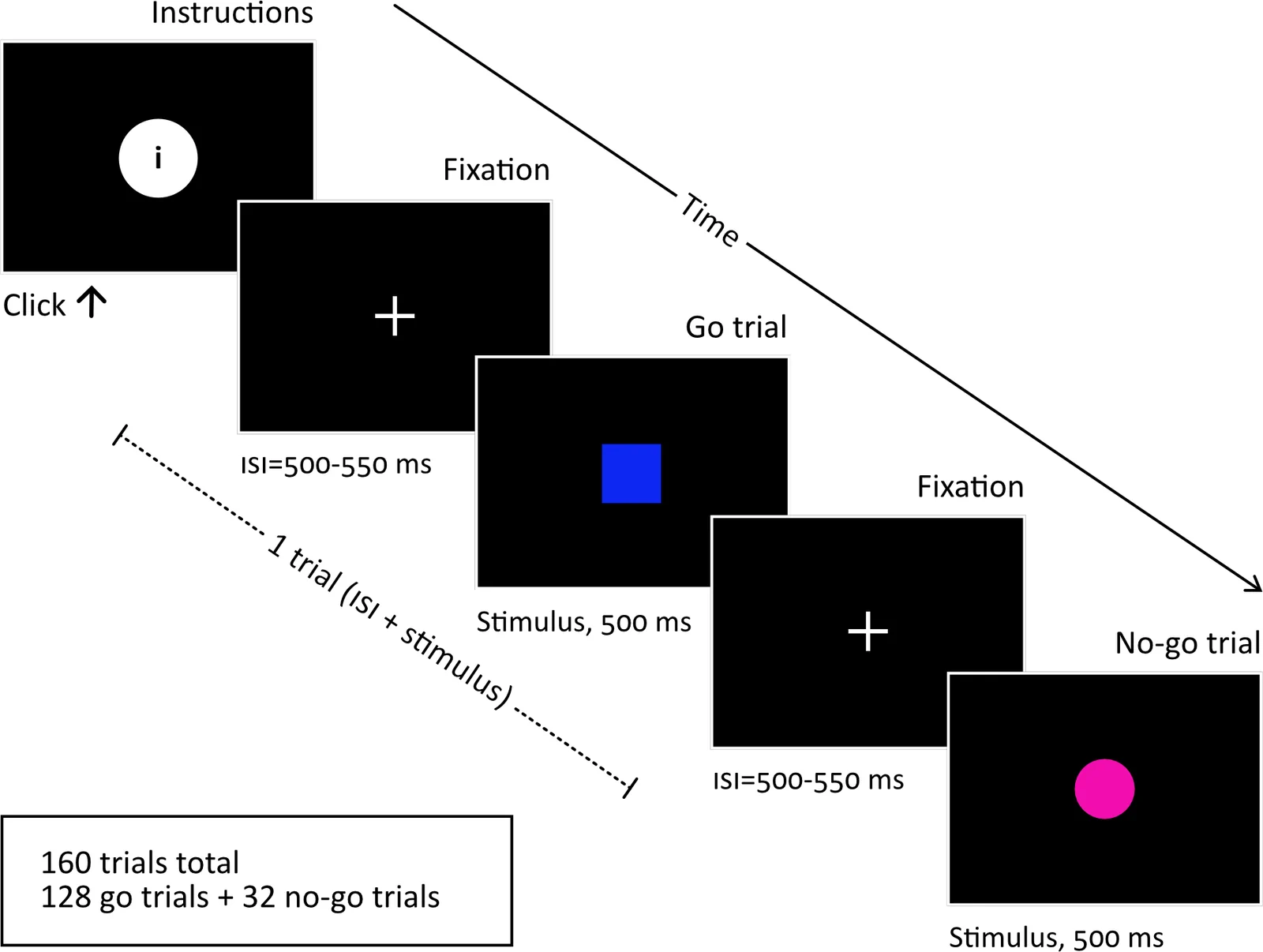
Schematic of the sequence of stimuli in the go/no-go task. The order of go and no-go trials will be randomized, and stimuli (color and shape) will be reversed in the posttest. ISI: interstimulus interval.

##### Onboarding

###### Intervention and Control Groups

Participants will be pseudorandomly assigned to either an intervention or a control group using the pretest measurements of IQ (RSPM), perceived health, behavioral performance, and cognitive measurements, to reduce differences between groups before the intervention (see details under *Randomization*).

After the participant is assigned to a group, the research team will schedule a 30-minute in-person onboarding session with the participant. In this session, participants will be instructed with the goals, expectations, and criteria for success in the healthy lifestyle intervention. Participants in the intervention group will be lent an Apple Watch Series 8 or 9 (Apple Inc) to be used throughout the intervention and returned when the intervention ends. Participants in the control group will receive a copy of the *Physical Activity Guidelines for Americans* (PAG) published by the US Department of Health and Human Services (Figure 5).

The onboarding for the intervention group will include steps inherent to the smartwatch use and setup: signing a complementary consent form, pairing their phone with the smartwatch, ensuring proper fit (use on nondominant arm, correct strap size, snug fit, and how to tighten during workouts), configuring goals, activating health and activity notifications, and sharing of data with the research team. Participants will be allowed to increase the goals configured but not to reduce them to a lower value than the initial setup. Participants will also be able to customize all other aspects of the smartwatch as if it was their own.

The control group will be guided through the outline of the PAG executive summary and they will be asked to read it in full and to ask any questions they might have. The PAG provides evidence-based advice for adults on how physical activity can help promote health, including specific guidelines that set the standard goals for our intervention [32]. Participants in the control group will receive a printed copy of the executive summary and a digital copy of the full 118-page document with an in-depth analysis and explanation of the guidelines. Participants will not be required to read the full document but will be encouraged to do so if they want to learn more about the intervention standards.

To be considered a successful intervention, both groups must achieve each of the following goals in at least 80% of the days:

- Stand and move for at least 1 minute during 12 different hours daily.
- Exercise at least 30 minutes of moderate aerobic activity daily (ie, activities that increase the heart rate such as brisk walking).
- Sleep at least 7 hours daily.

Participants must commit to achieving these goals. After the goals, expectations, and criteria are understood, we will schedule 4 weekly remote (video conference) check-ins with all participants. These check-ins will be scheduled on the same day and time for each week. After scheduling, participants will start the intervention.

**Figure 5.**
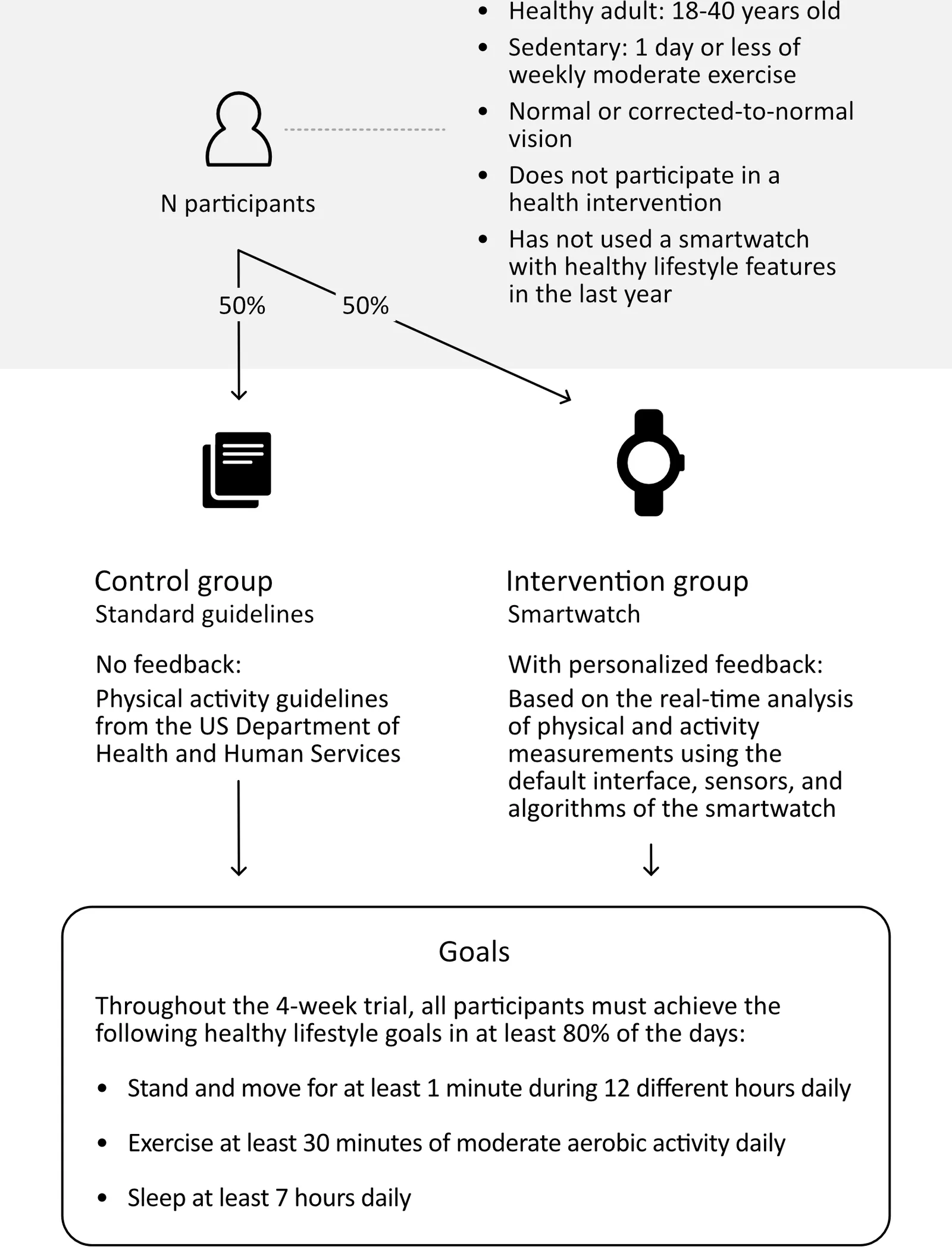
Overview of the intervention procedure, including goals.

###### Operationalization of Intervention Goals

The intervention goals outlined above are operationalized in the following way to ensure consistent measurement across groups (Table 1):

- Stand and move: it targets the interruption of prolonged sedentary behavior throughout the day. This goal requires participants to take active breaks of at least 1 minute during 12 different hours, which could reduce health risks associated with prolonged sitting during work or leisure time, independent of total exercise duration [33]. For the intervention group, the smartwatch will detect movement via accelerometer data and nudge participants if they have been sedentary in the first 50 minutes of each hour while awake. Participants in the control group will self-monitor and track their progression.
- Exercise: it targets an increase in moderate to vigorous physical activity daily. By engaging in at least 30 minutes of moderate-intensity aerobic exercise daily, participants will meet the weekly target of 150 minutes recommended for adults by the US Department of Health and Human Services [32]. Moderate-intensity aerobic activity is defined as sustained periods of physical activity that elevate heart rate, such as those performed at or above the intensity of a brisk walk. For the intervention group, the smartwatch will continuously monitor exercise intensity using photoplethysmography and accelerometer sensors. These sensors will measure the heart rate and movement, providing real-time feedback to participants on their progress. Participants in the control group will self-assess moderate intensity using the PAG definition of “anything that gets your heart beating faster counts” and keep track of their progression.
- Sleep: it targets following the sleep recommendations for adults. Participants must sleep 7 or more hours daily throughout the intervention to promote optimal health [34]. For the intervention group, the smartwatch will estimate sleep duration through motion and heart rate patterns using accelerometers and photoplethysmography sensors. Participants in the control group will self-monitor and track their total sleep duration.
- Success criteria: Achieving each goal on 80% of the days represents a meaningful adherence threshold of the intervention. Both groups will report the completion of goals during check-ins. Smartwatch data from the intervention group will be used to verify and discuss self-reported measurements.

**Table 1.** Operationalization of intervention goals.

Goal	Intervention group	Control group	Rationale
Stand and move: at least 1 minute during 12 different hours daily	Smartwatch: accelerometer detects movement in 12 different hours; nudges participants after 50 minutes of inactivity each hour while awake	Self-monitor and track progression toward hourly active breaks	Interrupting prolonged sitting reduces health risks independent of total exercise [33]
Exercise: at least 30 minutes of moderate aerobic activity daily	Smartwatch: PPG^a^ (heart rate) and accelerometer (movement) sensors continuously monitor exercise intensity; real-time feedback on progress; activities at or above brisk walk intensity count toward goal	Self-assess using the PAG^b^ definition: “anything that gets your heart beating faster counts;” self-track progression	Meets recommended target of 150 weekly minutes of moderate aerobic activity for adults [32]
Sleep: at least 7 hours daily	Smartwatch: motion (accelerometer) and heart rate (PPG) patterns estimate sleep duration	Self-monitor and track total sleep duration	Meets the recommendation for adults to promote optimal health [34]
Success criteria: achieve goals on at least 80% of the days	Smartwatch: continuous tracking; data verified and discussed during weekly check-ins	Self-reported during weekly check-ins	Meaningful adherence threshold for lifestyle interventions

a PPG: photoplethysmography.

b PAG: Physical Activity Guidelines for Americans.

### Intervention

The healthy lifestyle intervention will last 4 weeks. To validate the intervention success, all participants must achieve the required healthy lifestyle goals during this phase. They are also expected to complete each of the weekly check-ins. These remote check-ins will help us understand their experience with the intervention at different time points, track the completion of goals, and ask for significant issues they might be facing. Each check-in will consist of a short semistructured interview and a questionnaire and will be 10 to 15 minutes long. Check-ins will be audio-recorded and transcribed unless the participant requests us not to, in which case, notes will be taken. In the short interview, participants will be asked questions about their experience with the intervention, including issues, workarounds, helpful factors, their discoveries or usages, as well as any change(s) or customization they made to the intervention. The questionnaire consists of 6 questions to measure their progress toward completing the intervention (Table 2). Two questions will be asked for each goal (stand and move, exercise, and sleep). The first question asks how many days (0‐7) in the past week they successfully completed their goal. The second question asks for an average of partial completion on unsuccessful days (eg, average minutes of moderate aerobic activity exercise on days the 30-minute goal was not met). At the end of the third check-in, the last in-person posttest assessment will be scheduled right after the end of the intervention.

**Table 2.** Questionnaire questions and response scales.

Goal	Question 1	Response scale	Question 2	Response scale
Stand and move	“How many days could you complete the stand and move goal this week?”	0‐7 days	“Think of the days you could not complete your stand and move goals. On average, how many hours would you say you achieved out of 12?”	0‐11 hours
Exercise	“How many days could you complete the exercise goal this week?”	0‐7 days	“Think of the days you could not complete your exercise goals. On average, how many minutes would you say you achieved out of 30?”	0‐29 minutes
Sleep	“How many days could you complete the sleep goal this week?”	0‐7 days	“Think of the days you could not complete your sleep goals. On average, how many hours would you say you achieved out of 7?”	0‐6.9 hours

### Posttest

This final session will last approximately 60 to 90 minutes. The posttest follows the same assessment structure as the pretest but does not require the participant to provide contact information, complete the RSPM, or sign an additional consent form (Figure 1). Additional assessments (not included in the pretest) are as follows: the System Usability Scale (SUS), a semistructured interview, and the setting away of the device (intervention group only). The roles of the stimuli during the go/no-go task will be reversed for each participant to control for practice effects and color or shape bias from the pretest.

After completing the SF-36 and while the research team continues the EEG setup, participants will answer the SUS. The SUS is a widely used standardized questionnaire for the assessment of perceived usability [35-37]. The SUS includes 10 items (eg, “I thought the system was easy to use”) with responses on a Likert scale from 1 (strongly disagree) to 5 (strongly agree), resulting in a score range of 0 to 100. Participants will answer the SUS based on their experience while using the smartwatch (intervention group) or following the PAG executive summary (control group).

After completing the go/no-go task and removing the EEG electrodes and cap, there will be a short 5-minute break. The research team will then conduct a semistructured interview with questions about the participant’s experience, preferences, and feelings after completing the intervention. Interviews will be conducted by trained research personnel following a standardized script (Multimedia Appendix 1). Both the intervention and control groups will follow the same script with questions adapted to their specific intervention (eg, “experience using the smartwatch” vs “experience using the guidelines”). Interviews will be audio-recorded and transcribed for analysis unless the participant requests not to. The semistructured format allows for a consistent coverage of relevant topics, while providing enough flexibility to explore experiences specific to each participant. The assessment lead will conduct the interview, while a research assistant will take notes of the participant’s answers on a separate computer. The topics included in the interview are personal background and daily routine, intervention adherence and experiences, perceived benefits, and feedback and future considerations.

If the participant is in the intervention group, setting away of the smartwatch is included in the posttest. This step includes backing up and retrieving the health data gathered by the smartwatch, stopping data sharing with the research team, and unpairing the smartwatch from their personal devices.

### Measurements

#### Primary Outcome Measures

The primary purpose of this protocol is to assess the effectiveness of a smartwatch healthy lifestyle intervention on cognitive enhancement in healthy adults. Our primary outcome (cognitive function) is indicated by changes in executive function after the intervention, specifically inhibitory control.

Executive functions are top-down processes essential for goal-directed coordination of thoughts and behavior [17,38]. These functions can be enhanced through healthy lifestyle interventions [15]. We focus specifically on inhibitory control for several evidence-based reasons. Inhibitory control captures what is considered common among the 3 core executive functions (inhibition, updating, and shifting), with inhibition tasks showing no factor-specific variance, making them optimal for assessing executive function with minimal task impurity [39]. Moreover, inhibitory control is foundational for adaptive outcomes in healthy adults, supporting impulse management, attention focus, and the performance of other executive functions [40]. It is also predictive of academic success [41], as well as health and financial status in adulthood [42,43]. Furthermore, it has been proposed for its potential transfer effects, leading to generalizable cognitive benefits [44]. The go/no-go task elicits the ERPs N2 and P3, well-validated electrophysiological markers of inhibitory control in EEG literature [20,45]. Notably, previous research has demonstrated that N2/P3 ERPs are sensitive to short-term lifestyle interventions. Moreno et al [20] showed significant ERP changes after a 4-week intervention, providing a direct precedent for our methodology. Comparative integrity assessments of N2/P3 amplitudes before and after the intervention will be used to evaluate changes in inhibitory control.

#### Secondary Outcome Measures

In addition to our primary outcome, we assess secondary effectiveness outcomes and evaluate engagement and acceptability to gain a comprehensive understanding of the intervention’s impact on cognitive enhancement.

The complexity of evaluating a smartwatch healthy lifestyle intervention targeting cognitive enhancement requires a comprehensive, multidimensional assessment framework [18,19]. Furthermore, using multiple measures to assess the intervention’s impact increases the confidence in our findings. Consequently, we use a convergent parallel mixed methods approach (quantitative and qualitative) [46] to assess 3 essential, interdependent constructs (Figure 6):

- effectiveness, which refers to the impact of the intervention on cognitive function, behavioral performance, and perceived health and quality of life
- engagement, which we define as the participants’ interactions and commitment to the intervention
- acceptability, which refers to the participants’ perceptions and satisfaction with the intervention.

The constructs of engagement and acceptability are binding prerequisites to effectiveness, speaking to the sociotechnical complexity of wearable health intervention components [16,18,19]. Even if an intervention has the potential to enhance cognition, it will not occur if participants do not engage with it or find it unacceptable for continued use. This approach will provide us not only with a better understanding of what factors, such as behavior change techniques included in the smartwatch or unanticipated external factors, might affect the effectiveness and engagement of the intervention [47] but also indicators of why and for whom they work.

We further operationalize effectiveness in three secondary outcomes: (1) cognitive function (in addition to primary outcome), (2) behavioral performance, and (3) perceived health and quality of life.

The secondary outcome of cognitive activity is indicated by changes in power of each canonical frequency band (delta: 1‐4 Hz, theta: 4‐8 Hz, alpha: 8‐13 Hz, beta: 13‐30 Hz, and gamma: >30 Hz) of resting-state recordings. Resting-state gamma power is associated with cognitive performance [48,49]. Changes for each canonical frequency are compared across groups and time points. The outcome for behavioral performance is indicated by changes in average reaction time of correct go trials (in milliseconds), and accuracy of correct no-go trials for the go/no-go task before and after the intervention. The outcome for perceived health and quality of life is indicated by changes in all scores for the SF-36 before and after the intervention (scale: 0‐100).

Engagement outcomes are indicated by the percentage of goals reported as complete at each check-in point and reported averages of how much of the goals were completed on unsuccessful days. Acceptability outcomes are indicated by SUS scores after the intervention (scale: 0‐100).

Lastly, outcomes from semistructured interviews (weekly check-ins and posttest) include notes, audio recordings, and transcripts. Following our convergent parallel mixed methods approach, we will compare and interpret the themes identified side to side with quantitative outcomes. This comparison is expected to provide depth and a comprehensive understanding of factors influencing the intervention. While we anticipate finding themes related to all 3 constructs, we remain open to identifying unexpected items. More details are provided in the *Data Analysis* section.

**Figure 6.**
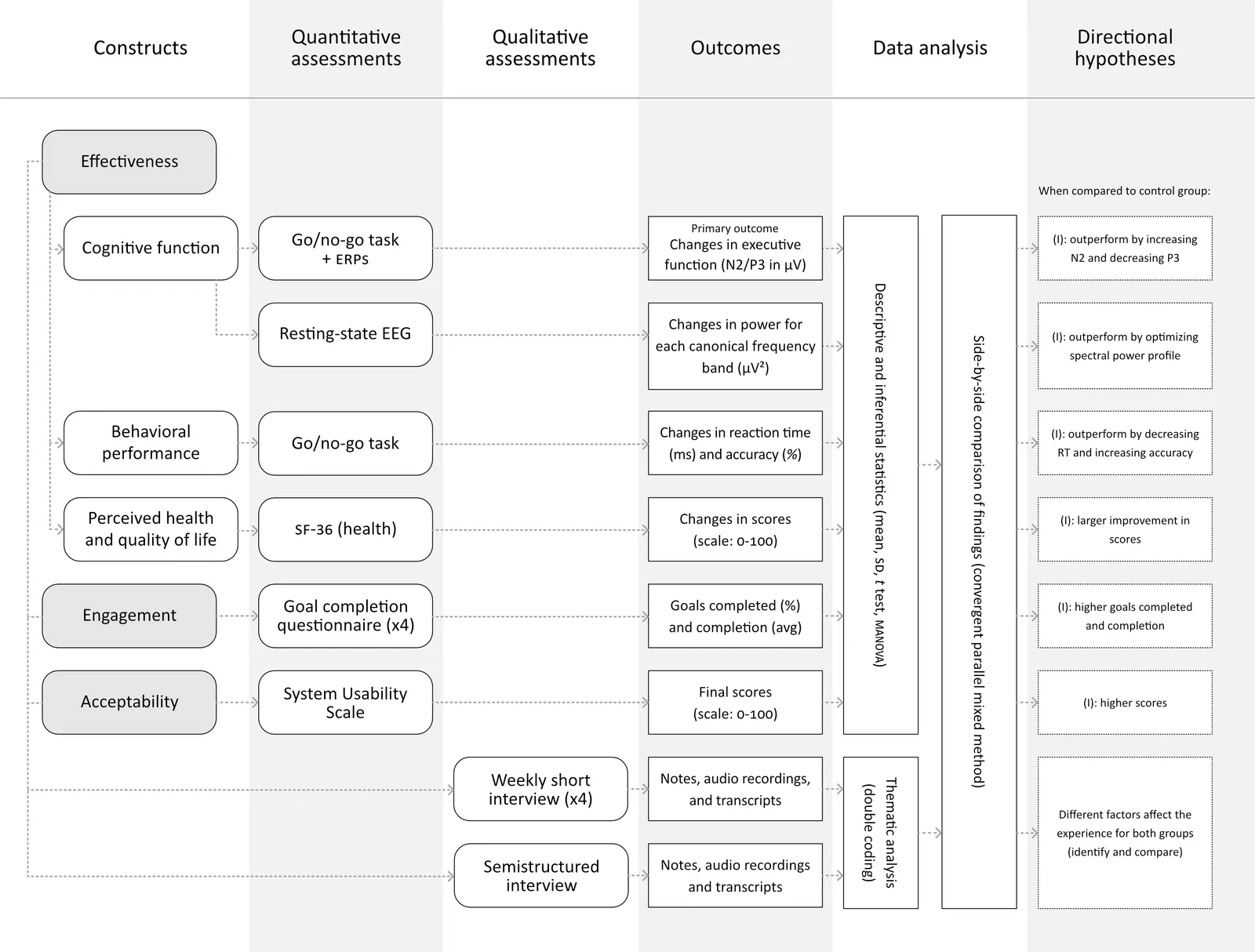
Overview of constructs with their corresponding assessments, outcome measures, data analysis, and hypotheses. avg: average; EEG: electroencephalogram; ERP: event-related potential; I: intervention group; MANOVA: multivariate analysis of variance; RT: reaction time; SF-36: Short Form Health Survey.

#### Exploratory Outcomes

In addition to the primary and secondary outcomes, we seek to explore interactions among the 3 constructs. Outcomes include descriptions of possible interactions between the constructs of effectiveness, engagement, and acceptability of a smartwatch with a healthy lifestyle intervention throughout the intervention. Engagement outcomes (goal completion) recorded by the smartwatch will be used for comparison with self-reported outcomes.

### Apparatus

#### Smartwatch

Participants in the intervention group will use either a GPS-enabled Apple Watch Series 8 or 9 (Apple Inc) during the intervention. Each cohort will use the latest operating system at the launch of the study (ie, watchOS 11.3.1). To standardize the features in the watch, user interface, and data collected, participants will be instructed not to update the watchOS version after the intervention begins. The smartwatch will facilitate the intervention using features such as real-time feedback and goal completion notifications. WatchOS includes an interface for the completion of goals called Activity Ring Goals comprising 3 daily goal elements: stand, move, and exercise. This interface will be set up using our intervention goals (stand: 1 minute during 12 different hours daily, exercise: 30 minutes daily). In addition, we will set up a goal of 7 hours of sleep in the health app, and default visual and haptic notifications related to the completion of these goals will be activated. Apple Watch was chosen because of its widespread adoption [26], which could provide an integral snapshot of the current impact of wearables in cognitive function.

#### EEG

The EEG recordings will be conducted using a BioSemi Active Two system (BioSemi BV) with pin-type and flat-type active electrodes. In total, 64 pin-type electrodes will be placed in the standard 10‐20 international system configuration using a BioSemi head cap. Also, 2 pin-type electrodes will be used for CMS and DRL. Five flat-type electrodes will be used for EOG and mastoid locations (See *Procedure* for details). Different head cap sizes will be available for head circumferences ranging from 50 cm to 62 cm.

The system consists of 2 laptops (one for visual stimuli and another for raw data visualization and recording), respective chargers, mouse, and the BioSemi system. The stimulus laptop will use Presentation (NeuroBehavioral Systems, Inc), a stimulus delivery and experiment control program for neuroscience. This software allows the coding of the resting-state and go/no-go tasks while also allowing the recording of event codes for data analysis. Event codes are recorded with submillisecond temporal precision for stimulus delivery and responses given by the participants. The raw data laptop will use BioSemi ActiView software, a comprehensive acquisition program designed to display all channels and save the data in BDF format. The sample rate will be set to 512 Hz. Finally, the BioSemi system includes an analog-to-digital box, a battery box and charger, active electrodes, head caps, USB receiver, cables, conductive gel, plastic syringes, adhesive discs, alcohol wipes, and towels.

Due to the vulnerability of EEG signals to high noise-to-signal ratio, all data acquisition procedures will be performed in a highly controlled environment [50]. Participants will be isolated in a noise-shielded room with controlled lighting conditions. Participants will also be instructed to avoid moving or blinking to reduce discarded data and increase the signal-to-noise ratio.

### Data Analysis

#### EEG Data Processing

Figure 7 shows the EEG-ERP data processing pipeline. EEG data will be preprocessed using EEGLAB (version 2023.0) [51]. ERP data will be extracted using ERPLAB (version 9.20) [52]. Both toolboxes require the installation of MATLAB (version R2023a; MathWorks).

**Figure 7.**
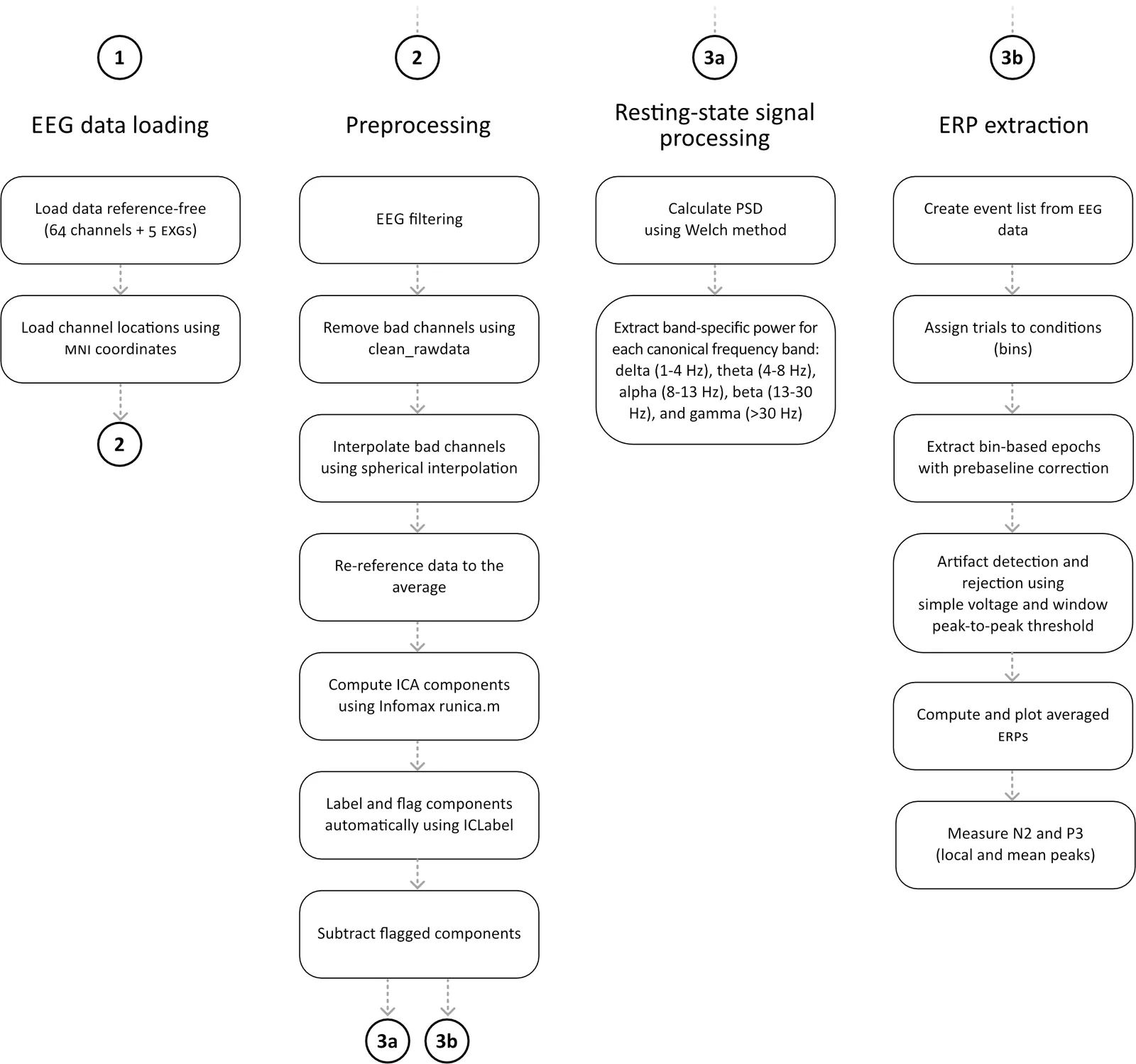
Visual representation of the EEG-ERP data processing pipeline. EEG: electroencephalogram; ERP: event-related potential; EXG: external electrode; ICA: independent component analysis; MNI: Montreal Neurological Institute; PSD: power spectral density.

##### Data Loading

EEG data will be loaded reference-free from the BioSemi BDF file using the BIOSIG toolbox embedded in EEGLAB. Channel locations will be loaded according to the 64-channel 10‐20 system, using corresponding Montreal Neurological Institute coordinates.

##### Preprocessing

The EEG signal will be filtered using a low pass of 30 Hz (go/no-go task EEG) or 80 Hz (resting-state EEG) and a high pass of 0.5 Hz [53], a roll-off of 24 dB per octave, and fourth-order infinite impulse response Butterworth filter [54]. All 64 scalp channels and 5 external electrodes (3 EOG electrodes and 2 on mastoids) will be included. Bad channels will be removed using the EEGLAB plug-in clean_rawdata with a high-frequency noise SD threshold of 4 and channel correlation removal of 0.8 [54]. Removed channels will be interpolated using spherical interpolation. Then, data will be re-referenced to the average. Independent component analysis will be performed using the Infomax runica.m algorithm. We will then use ICLabel for automated component labeling and to flag eye and muscle components with a minimum threshold of 0.9 [54]. Flagged components will then be subtracted from the dataset.

##### Resting-State Signal Processing

Spectral power analysis will be conducted on the preprocessed resting-state EEG data. Power spectral density will be computed using the Welch method for each channel across canonical frequency bands: delta (1‐4 Hz), theta (4‐8 Hz), alpha (8‐13 Hz), beta (13‐30 Hz), and gamma (>30 Hz). Band-specific power metrics will be statistically compared across groups (intervention vs control) and time points (preintervention vs postintervention).

##### ERP Extraction

First, we will create an event list (EventList) from the EEG data. Then, we will assign each trial to one of four predefined bins: (1) correct go trial, (2) incorrect go trial, (3) correct no-go trial, and (4) incorrect no-go trial. After bins are assigned, we will extract bin-based epochs with a time range from −200 to 800 milliseconds after the stimulus was presented and apply prebaseline correction. Artifact detection and rejection on epoched data will be done using a combination of a simple voltage threshold of ±200 µV to detect large drifts in voltage and a window peak-to-peak threshold of 150 µV to detect artifacts involving sudden voltage changes [55]. The percentage of trials discarded should be lower than 10%. Finally, average ERPs will be computed and plotted. The N2 and P3 components of the ERP will be measured from correct go and no-go trials (bins 1 and 3, respectively). Both the maximum voltage (local peak) and the average voltage (mean peak) will be measured for both components between 2 specific time points. Based on established literature, the N2 component will be measured between 250 and 350 milliseconds post stimulus and the P3 component will be measured between 300 and 500 milliseconds post stimulus, both at frontocentral electrode clusters, consistent with standard windows and electrode sites reported in the go/no-go ERP literature [56]. Visual inspection of grand average ERPs will be conducted to verify that these windows appropriately capture the components in the dataset.

### Data Analysis Plan

#### Approach and Tools

Our convergent parallel mixed methods approach will involve the analysis of multiple sources of data through quantitative and qualitative methods. Descriptive and inferential statistics will be used to evaluate the hypotheses, while a thematic analysis will be used to uncover factors that might affect the participant’s experience throughout the intervention. This analysis will be done using a combination of tools: JMP, a computer software for statistical analysis (version 16.1; JMP Statistical Discovery LLC); Python scripts for statistical analysis (version 3.13.2; Python Software Foundation); R software (version 4.4.1; R Foundation for Statistical Computing), using the *afex* package [57]; and NVivo, a computer software for data analysis (version 14; QSR International Pty Ltd).

#### Evaluation of Primary and Secondary Criteria

We evaluate the effectiveness, engagement, and acceptability of the smartwatch intervention for cognitive enhancement. The research questions (RQs), quantitative hypotheses (H), and qualitative components (Qual) are organized by these three constructs:

1. RQ1 (effectiveness): how does a smartwatch (with/without) healthy lifestyle intervention enhance cognitive function (primary), behavioral performance, and perceived health over time?

- H1.1—primary criterion (cognitive activity—ERP analysis): the intervention group will show greater improvement in electrophysiological markers of inhibitory control by increasing N2 amplitudes and decreasing P3 amplitudes compared to the control group after the intervention [20].
- H1.2 (cognitive activity—power analysis): the intervention group will show an optimized spectral power profile post intervention compared to the preintervention spectral power of the intervention group and spectral power of the control group at all time points.
- H1.3 (behavioral performance): the intervention group will outperform the control group in decreasing reaction time, and increasing accuracy in a go/no-go task compared to the control group after the intervention.
- H1.4 (health perception): the intervention group will report a larger improvement in SF-36 scores compared to the control group after the intervention.
- Qual-1: identify factors affecting perceived changes in cognition, behavior, and perceived health

1. RQ2 (engagement): how engaging is a smartwatch among healthy adults throughout a healthy lifestyle intervention?

- H2: the intervention group will report a higher number of completed goals and higher averages of completion on unsuccessful days compared to the control group.
- Qual-2: identify barriers, facilitators, strategies, and patterns of use

1. RQ3 (acceptability): what factors influence how acceptable a smartwatch is among healthy adults during a healthy lifestyle intervention?

- H3: the intervention group will rate the wearable higher in SUS scores compared to the control group guidelines.
- Qual-3: identify factors affecting usability, satisfaction, and preferences

Each research question will be addressed through both quantitative and qualitative methods (Figure 6). Quantitative analyses will provide measurable outcomes, while qualitative analyses will provide depth, context, and potential explanations of why these outcomes are observed and what factors could influence them.

#### Quantitative Data Analysis

Multivariate analysis of variance (MANOVA; α=.05) will be used to test hypotheses where the effect of interest includes multiple independent variables (H1.1, H1.2, H1.3, and H2). H1.1 will examine the effect of group (intervention or control; between-subjects), assessment (A or B; within-subjects), condition (correct go or correct no-go trial; within-subjects), and electrode (within-subjects) on ERP amplitude. H1.2 will examine the effect of group (intervention or control; between-subjects) and assessment (A or B; within-subjects) on canonical frequency band power. H1.3 will examine the effect of group (intervention or control; between-subjects), assessment (A or B; within-subjects), condition (correct go or correct no-go trial; within-subjects), and electrode (within-subjects) on reaction time and accuracy. Finally, H2 will examine the effect of group (intervention or control; between-subjects) and assessment (A or B; within-subjects) on goal completion.

Independent samples *t* tests (1-tailed) will be used to test hypotheses where the primary effect of interest is intervention type (group) on a single dependent variable (health perception for H1.4 and SUS scores for H3).

To account for multiple comparisons, false discovery rate correction will be applied across quantitative outcome measures where appropriate. For EEG and behavioral outcomes, electrophysiological markers (N2/P3 amplitudes) and behavioral measures (reaction time and accuracy) will be treated as complementary indices of a single underlying cognitive construct (inhibitory control) rather than independent hypothesis tests, consistent with standard practice in ERP literature [20]. Bonferroni-corrected pairwise *t* tests (1-tailed) will be conducted if the main model is significant, to localize effects. Effect size (η^2^) will be reported.

One assumption is that if healthy adults in the intervention group perform better in effectiveness tests after the intervention (at posttest) compared to the control group, then the smartwatch had a larger positive impact. Any other improvement that occurred would have randomly occurred in their everyday activities.

#### Qualitative Data Analysis

Recorded interviews at each check-in and after the intervention will be analyzed using thematic analysis [58,59]. We will implement a 6-phase approach to identify factors (themes) that affect the intervention’s experience (Figure 8). Transcripts of recordings will be initially automated and then manually verified for accuracy, including for nonlatent cues in responses. All interviews will be double-coded independently by different research team members to ensure reliability [46,60]. Coding agreement will not be measured, as it could hinder valuable insights emerging from disagreements and discussion [22,61]. Disagreements will be discussed and the coding framework refined among the research team throughout the coding process [62]. Codes identified in transcripts will be triangulated with codes identified live by trained research personnel during each interview. Live coding is implemented as a tool to preserve the context and intent of responses potentially lost in audio recording and transcriptions [63,64]. Themes within data will be identified using a data-driven inductive method based on the grounded theory method. This method facilitates the discovery of insights specific to the intervention that could be potentially missed by deductive or theory-driven approaches [64,65]. Relevant themes will be then used to interpret the constructs of effectiveness, engagement, and acceptability of the intervention while allowing the discovery of other relevant factors. Analysis will be conducted separately for the intervention and control groups while maintaining time points to identify themes specific to group and time and then will be compared to identify differences in user experience between smartwatch- and guideline-based interventions.

**Figure 8.**
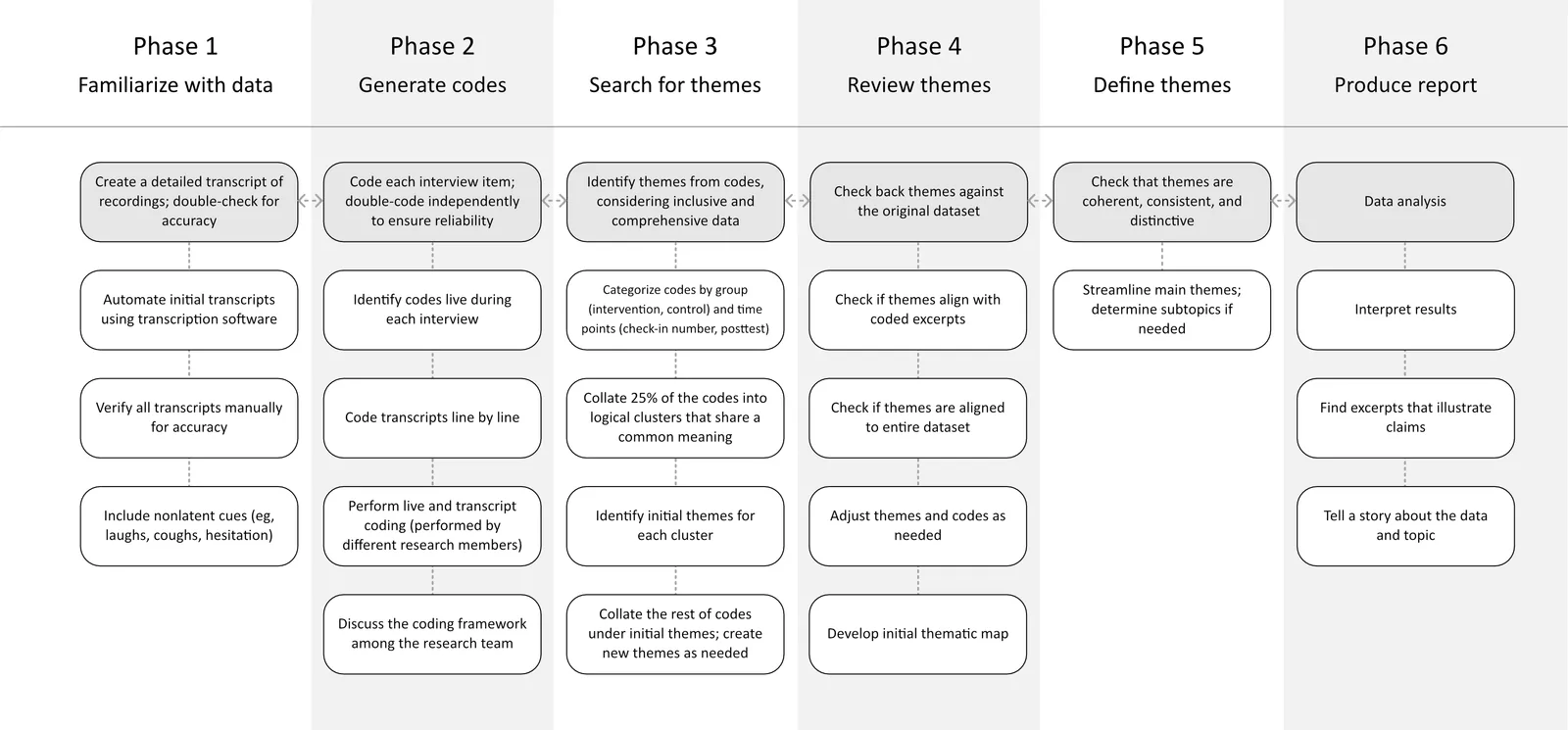
Visual representation of the phases of thematic analysis.

#### Integration of Quantitative and Qualitative Findings

We will use a convergent parallel mixed methods design to integrate and interpret quantitative and qualitative data [19,46]. The mixed methods design will follow the steps below (Figure 9):

1. Independent analysis: quantitative and qualitative analyses will be performed separately using their respective methods for each construct (Figure 6).
2. Joint display: results from both analyses for each construct will be presented in parallel to compare and relate outcomes.
3. Interpretation: comparison will be used to identify areas with
  - convergence: quantitative and qualitative findings support the same conclusions (eg, high SUS scores + positive usability feedback),
  - divergence: findings suggest contradiction and require further exploration (eg, improved cognitive outcomes but negative experiences), and
  - expansion: qualitative findings provide depth and context to quantitative outcomes (eg, *why* goal completion increased over time).
4. Exploratory outcomes: we seek to identify interactions between all 3 constructs. In addition, the intervention group’s engagement outcomes (goal completion) recorded by the smartwatch will be compared with self-reported goal completion, and differences will be reported.

**Figure 9.**
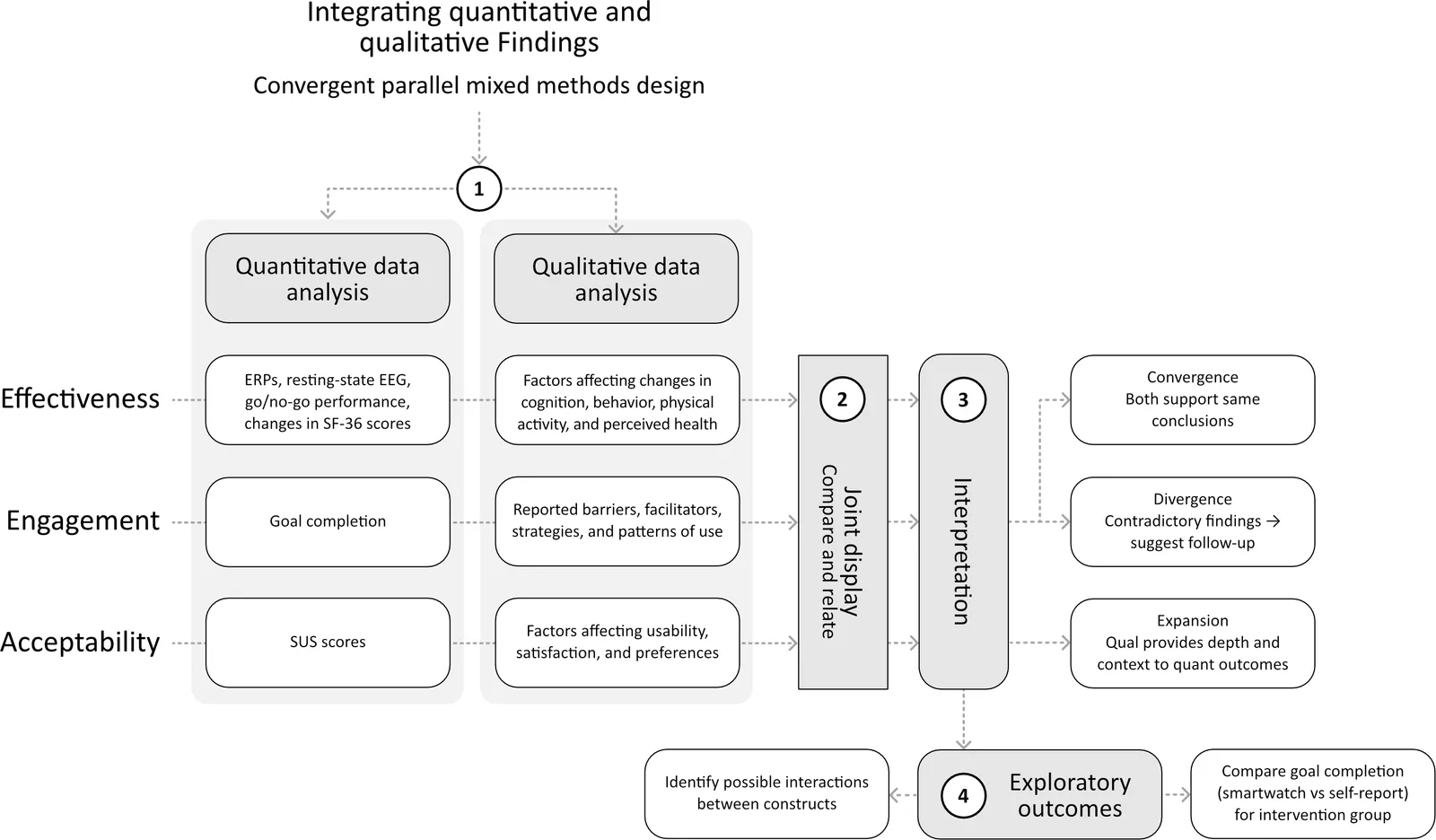
Visual representation of the convergent parallel mixed methods design. EEG: electroencephalogram; ERP: event-related potential; Qual: qualitative; Quant: quantitative; SF-36: Short Form Health Survey; SUS: System Usability Scale.

### Data Management

Personal information will be collected but not preserved. Information such as age, gender, job role, test results, EEG data, and interview recordings will be collected and used for analysis. Data retrieved will be deidentified. All the collected data will be coded, and a master list will be used while the study is ongoing. The master list will be deleted once the data are published. Digital data will be securely stored in firewall-protected servers at Simon Fraser University. Physical data will be securely stored in a laboratory location with restricted access to personnel involved in this study.

## Results

Participants are enrolled in the study using a continuous enrollment process. The first cohort to evaluate the feasibility of the study was conducted from March 2024 to September 2024, while the second cohort started enrolling participants in June 2025. As of October 2026, data have been collected from 48 participants. Currently, the data from the first cohort (n=24) are being processed and analyzed. The first results are planned to be published in early 2027.

## Discussion

### Expected Findings

Wearable technologies are expected to exponentially grow in everyday use [2] and have been found to be as effective as psychotherapy or pharmacotherapy treatments in supporting brain health [66]. Therefore, we anticipate a growth of research on their implementation during everyday activities. To our knowledge, this protocol presents the first comprehensive framework for evaluating the effectiveness, engagement, and acceptability of consumer smartwatch healthy lifestyle interventions targeting cognitive enhancement in healthy adults through randomized controlled comparison with integrated neurophysiological (EEG), behavioral, and experiential assessments. Effectiveness will be assessed through cognitive function (primary outcome: inhibitory control via EEG-based ERPs), behavioral performance, and perceived health. All 3 constructs will be evaluated through quantitative and qualitative assessments using a convergent parallel mixed methods design. This methodological integration addresses a critical gap in HCI wearable research, which frequently relies on single-arm observational studies focused on usability and adherence [16] without assessing cognitive outcomes or using controlled comparison. Our randomized controlled approach with an active control group enables us to determine if observed cognitive effects can be attributed to smartwatch-specific features or to general lifestyle modifications.

Due to the complex and interdisciplinary task of evaluating, designing, and developing cognitive enhancement interventions delivered through wearable technologies for everyday use [6,67], there is an increased need for frameworks that assess not only effectiveness (does it enhance cognition?), but also engagement (do people use it?) and acceptability (do people find it satisfactory?) [19]. Furthermore, without controlled comparison, HCI studies on wearables cannot distinguish device-specific features from general behavior change [16]. In this protocol, we propose a convergent parallel mixed methods study that evaluates effectiveness, engagement, and acceptability using a combination of quantitative and qualitative measurements and analysis within a randomized controlled design. Our measurements make use of established instruments, questionnaires, tasks, and EEG as a brain imaging method. We detail procedures for the implementation and adaptation of EEG, including data processing and statistical analysis.

As suggested by our directional hypotheses, we anticipate that participants using a smartwatch in the intervention group will outperform participants in the control group in all 3 constructs of effectiveness, engagement, and acceptability. Furthermore, we expect our qualitative findings to reveal how factors identified in interviews influence the cognitive enhancement outcomes measured in our quantitative evaluations [14]. With this integrated understanding of effectiveness, engagement, and acceptability we seek to inform the design of future wearable cognitive enhancement interventions.

### Limitations

We acknowledge the inherent limitations of our proposed study design. Our protocol is limited to a 4-week intervention. Significant changes in cognition and behavior have been demonstrated for this duration [20], and it is short enough to keep pace with innovation in the field [48] but still could be influenced by a novelty adoption effect [11]. Novelty adoption effects decrease over time and are not indicative of long-term impact [68]. In addition, our implementation of weekly check-ins could affect the commitment of participants compared to regular at-home use, potentially influencing results. Future studies could consider implementing a follow-up stage to evaluate 2 factors: the intervention’s effect and experience after continued use or the duration of its effect after no longer having access to the intervention. Finally, our current setup evaluates the default healthy lifestyle intervention included on a widely commercially available smartwatch, the Apple Watch. Although this approach represents a valuable snapshot of how this specific technology is impacting cognitive function, it is recommended to proceed with caution when generalizing findings to other wearables, as the sensors, algorithms, and user experience are not standardized across devices and evolve rapidly over time [6]. Therefore, the evaluation of different devices, as well as the design and development of applications specifically tailored for brain health based on the findings of this study, is recommended.

### Conclusion

Our approach contributes to the field of HCI by providing a comprehensive framework for evaluating the effectiveness, engagement, and acceptability of wearable interventions targeting cognitive enhancement in healthy adults. Through randomized controlled comparison with integrated neurophysiological (EEG-based ERPs), behavioral, and user experience assessment, we will evaluate effectiveness through cognitive enhancement (inhibitory control), behavioral performance, and perceived health while systemically assessing engagement and acceptability factors through mixed methods analysis. In contrast with HCI studies evaluating wearables that rely on single-arm observational designs, our randomized controlled approach with an active control group allows us to determine whether observed cognitive effects can be attributed to smartwatch-specific features or to general lifestyle modifications. This framework provides HCI researchers and wearable developers with replicable guidelines for rigorously evaluating the effectiveness, engagement, and acceptability of consumer wearable technologies targeting the enhancement of cognitive function, potentially shaping the future of evidence-based wearable health interventions for healthy adults.

## Supplementary material

10.2196/81490 Multimedia Appendix 1 Script for all protocol phases: pretest, onboarding, intervention, and posttest.

10.2196/81490 Multimedia Appendix 2 Samples of the 36-item Short Form Health Survey (SF-36) instrument.

## Abbreviations

CMS: common mode sense
DRL: driven right leg
EEG: electroencephalogram
EOG: electrooculogram
ERP: event-related potential
H: quantitative hypothesis
HCI: human-computer interaction
ISI: interstimulus interval
MANOVA: multivariate analysis of variance
PAG: Physical Activity Guidelines for Americans
Qual: qualitative component
RQ: research question
RSPM: Raven Standard Progressive Matrices
SF-36: 36-item Short Form Health Survey
SUS: System Usability Scale
