# Supplementary material for: Assessing the Effectiveness, Engagement, and Acceptability of a Smartwatch Healthy Lifestyle Intervention for Cognitive Enhancement in Healthy Adults: Mixed Methods Electroencephalography Protocol
Source: JMIR Res Protoc. 2026 Nov 6;15:e81490. doi: 10.2196/81490 (PMC13753865; doi:10.2196/81490)
Supplement: Supplementary file 1 [file resprot-v15-e81490-s001.pdf]

STUDY SCRIPT (PRE)

"Healthy Lifestyle Intervention"

[Before the participant arrives, show slide with the study title.]

Greet the participant and welcome them to the lab. Remind them that if they want to go to the bathroom they should go now, as afterward it might be complicated because of the required set-up. Ask them to sit down in front of the stimulus computer and align the chair to the marks on the floor. Check that the center of the computer screen is leveled to their eyes, otherwise make necessary adjustments. Request them to turn off their phones or any device or notification that might distract them during the assessment. Ask them if they are comfortable, then proceed. Have a timer at hand to record the length of each procedure.

Consent

[Change to slide: consent form.]

Once the participant is sitted, hand them the consent form and say:

> "Since this is a research study, you must read and sign this consent form to perform all the required tasks. It explains the study's goal, the type of personal health information we will compile, and the tasks you will be asked to do. It also says that all the information obtained throughout this study will be kept confidential, and if you'd like to refuse or withdraw from it at any time, you can do it."

After they finish reading or signing the consent form, ask them the following questions:

> "Do you have any questions, or would you like any additional details?"
>
> *[Answer questions.]*

After signing the consent form, check that no fields are missing and hand them a copy for their records. Then, proceed to request contact information.

Contact Information

[Change to slide: contact information.]

Once the slide is shown, hand them the contact information form and say:

> "We will need to request some contact information from you. This information will be used to contact you and follow up with any topic related to the intervention. As mentioned in the contact form, this information will be destroyed whenever the results are published."

Once the form is filled, assign and write the participant ID at the bottom. This ID will be used from now on to store any assessment data for the participant. After assigning an ID, proceed to the first assessment.

Raven's Progressive Matrices

[Change to slide: RSPM Test.]

Show the participant the slide with the Raven's Progressive Matrices sample and say:

> "This is an observation and clear-thinking test. This task will be done on the laptop. We will go through an example before starting. Please click on the 'Click here to begin test' button to open the RSPM test form. [Wait for the form to open]. Please first input your participant ID [Let the participant know their ID]. Now let's go through the example together. [Point to the sample in the slide] As you can see, at the top part of this sample, there is a pattern with a bit cut out. First, you must think about the piece needed to complete the pattern correctly. Then find the right piece out of the six options shown below. Once you know the answer, select the checkbox corresponding to the piece that you think fits the pattern best. So, in this case, the correct answer would be... 3."

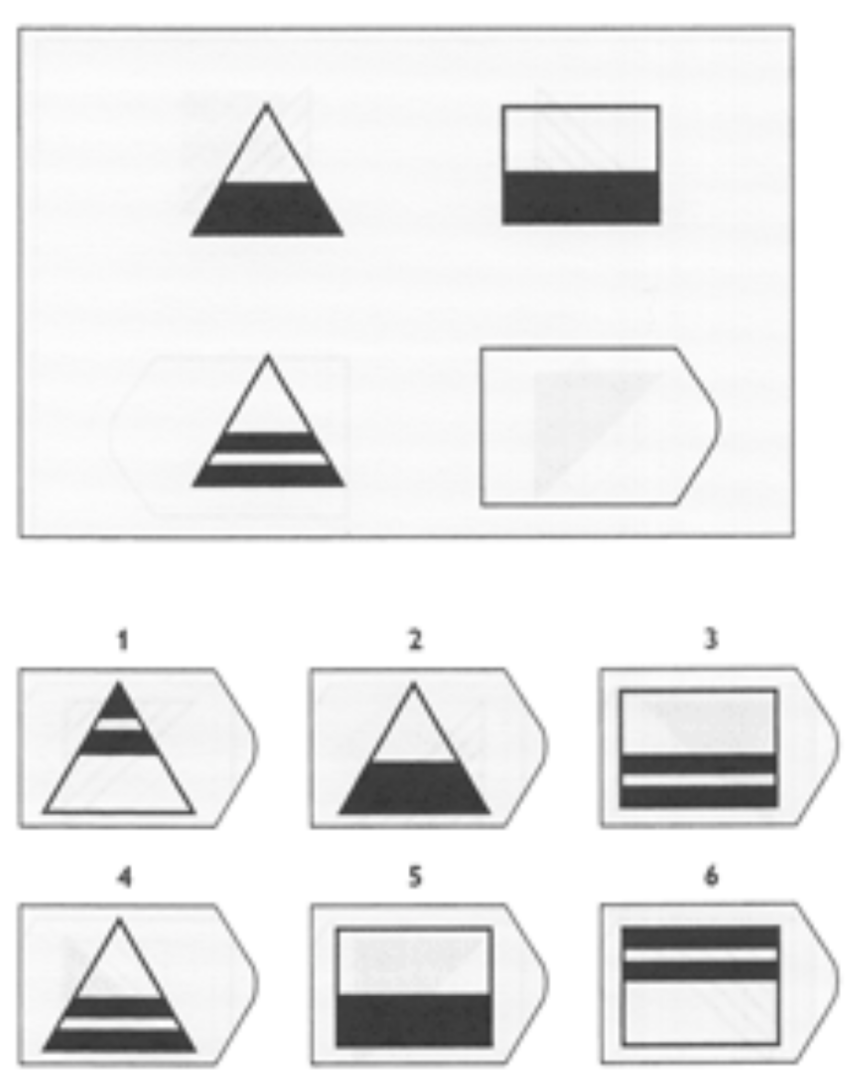

Ask if they understood

> "As I said earlier, you'll be completing this test on the laptop. You'll have to select the checkbox corresponding to the answer from 1 to 6, depending on which answer you think is correct. There is no time limit for this test, but we encourage you to finish it as fast as you can. You can now start whenever you're ready. Please let us know you have finished."

Start a timer. Stay around until the participant has finished in case there are any issues. Whenever the participant has finished, stop the timer, make a registry of the time, and then go back to the Assessment A slides. Ask them again if they wish to go to the bathroom before proceeding.

SF-36 quality of life (Do this while capping)

[Change to slide: EEG Capping.]

While capping, try to explain some of the steps to the subject. Keep them engaged and not bored. It is not good if they're bored before the tasks start. Say:

> "We want to pick up some of the electricity that your brain gives off. To do so, we will put this cap on your head. It will feel just like a swimming cap. We will start by placing two electrodes on your temples (point on your face), one beneath your left eye (point on your face), and two on your mastoids (point again on your neck). We do this to measure electrical activity given by the muscular movements around your eyes and neck. We will have to clean the area to get a better signal. We will also do some measurements of your head to select a right-sized cap."

Do it

Please remember to:

- Clean the temples and mastoids with an alcohol swipe (or an existing alternative);
- Measure the circumference of the head (the measuring tape must go over the inion and the nasion). This will allow you to choose the right-sized cap (if someone is on the limit, always choose the smallest of both options). Make a record of the selected size;
- Glue circular stickers onto external electrodes (1-5) with the center of the sticker coinciding with the center of the electrode and add some gel to the area;
- Put external electrodes 1 on the left temple, 2 on the right temple, 3 below the left eye (1.5 cm below the eyelid), 4 on the right mastoid, and 5 on the left mastoid. Remember to try to put these on with the wires directed to the back of the head so that they can be tucked behind the subject's ear;
- Put the cap on the subject's head. Ask them to help by holding the Fp1, Fpz, and Fp2 electrodes against her forehead. (Directions below)

> "Now, we'll put the cap on you, and we will put some gel into these holes (show the holes) because the electrodes need a medium to conduct the electrical signal from the scalp to the metal (show them how). I'll need your help. I'll need you to hold this front part against your forehead, please. (Place the three frontal electrodes of the cap in the correct place and let them hold it down while you pull the back part of the cap down – be sure that the cap is well applied by verifying the midline both longitudinally and transversally)."

[Change to slide: Health Survey.]

Show the volunteers the SF-36 questionnaire and say:

> "While we finish setting up, we would like to understand better how you feel, how well you can do your usual activities, and how you rate your own health. To help us better understand these things about you, we will ask you some questions about your general health. The survey will be done on the same laptop. Please click on 'Click here to begin test.' (Wait for the survey to open) This is not a test, and there are no right or wrong answers. Choose the response that best represents the way you feel. Please answer every question. As we proceed, please feel free to ask me any questions you may have."

While continuing to cap, please remember to:

- Start by gelling all the labeled electrodes.
- Insert the electrodes in the cap (two sets of 32 electrodes (A and B)). Be careful so that none of the electrodes touch metallic surfaces, and remember that they are very fragile (that's why we call them "angel hair"). It might be easier to put the electrodes around your neck so that there's not a lot of weight on them.
- Finally, insert DRL and CMS electrodes.
- Test the EEG recording signal.

After having a good signal from every electrode, proceed to the next task.

RestingState (5min)

[Change to slide: Resting State Recording.]

Say:

> "We are going to start with a simple task. This task aims to measure electrical activity in your brain while resting. Once it begins, a cross will appear in the center of the screen. You just have to focus on it for one minute. Please try not to move or blink too much. You can reread the instructions when we set up the recording."

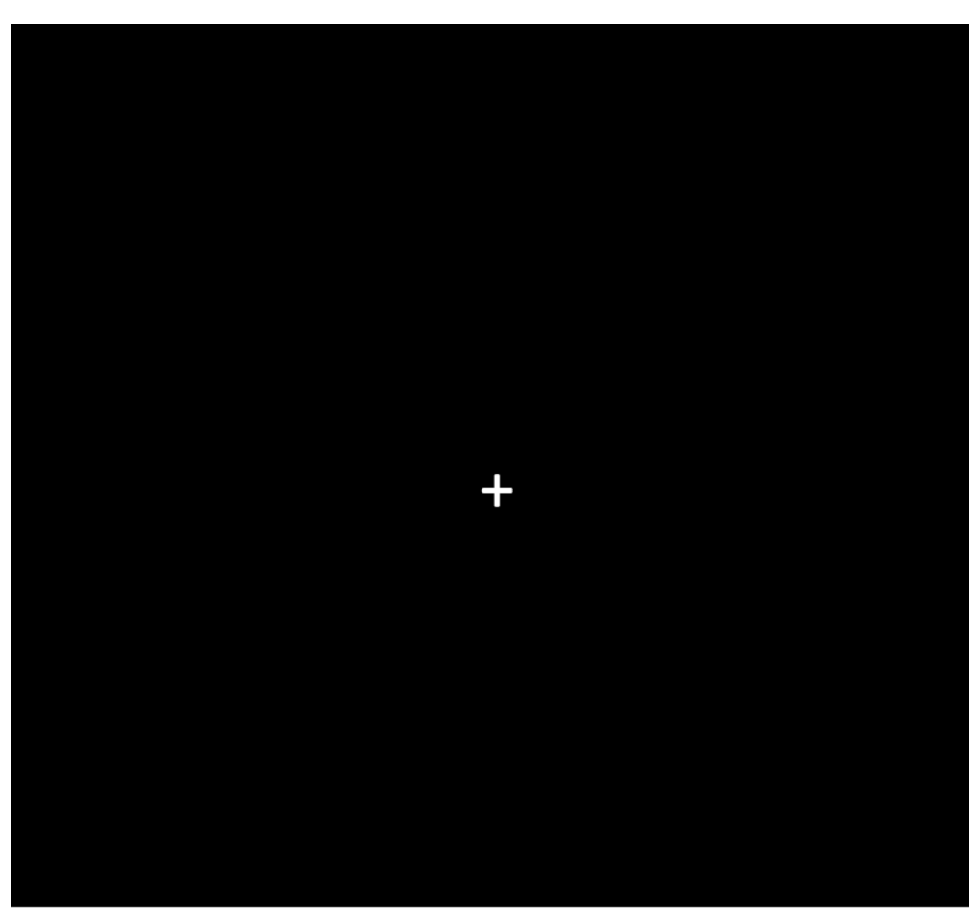

Open the Presentation files on the PRESENTATION laptop to open the Resting State task.

CNRL_SW_Presentation > Resting State > Resting State.sce

Important: When the Presentation window opens, check if the port codes are working before running the experiment. If they are not, you cannot see any events when you analyze the data. To do this, go to:

Settings > Port > Select Output Port 1 > Test > Send

You should be able to see the response (1) underneath the data acquisition window on the EEG RECORDING laptop.

Important: To save the EEG acquisition, you must press Start File and then Pause Save on the EEG RECORDING laptop. A window will appear where you can choose a name for this file (e.g., PA-07_RestingState_A) and write a short description of the task (e.g., Assessment A). Also, ensure that you select the checkbox for the 8 External electrodes and the 64 Regular electrodes. It will only save the recordings when the Pause Save button is green! Once the task is over, press Pause Save again and Stop (at the top/left of the screen) so that you can start a new file for the next experiment.

Once the output ports are working and you have started the recording, return to the Main tab and press Run to run the experiment. Then click Run Scenario for the instructions to appear full-screen on the screen. Now say:

> "After you complete each task, a new set of instructions will appear. Please read them carefully before proceeding. When you are ready, please click anywhere on the screen to begin."

When the minute has passed and the instructions for the second task appear on the screen, say:

> "We have finished the first task. Now, we will do another one-minute recording, but this time, we need you to close your eyes during the recording. Try not to move your eyes or your head during the recording. We will let you know when you can open your eyes again. When you are ready, please click anywhere on the screen to begin."

When the minute has passed, indicate the participant to open their eyes and thank them for following indications. Now, proceed to stop the recording on the EEG RECORDING laptop and finish the experiment on the PRESENTATION laptop.

Go/No-Go (10 min)

[Change to slide: Go/No-Go Task.]

Open the Presentation files on the PRESENTATION laptop to open the Go/No-Go task.

CNRL_SW_Presentation > Go-NoGo > Go-NoGo Task > Go-NoGo Task.exp

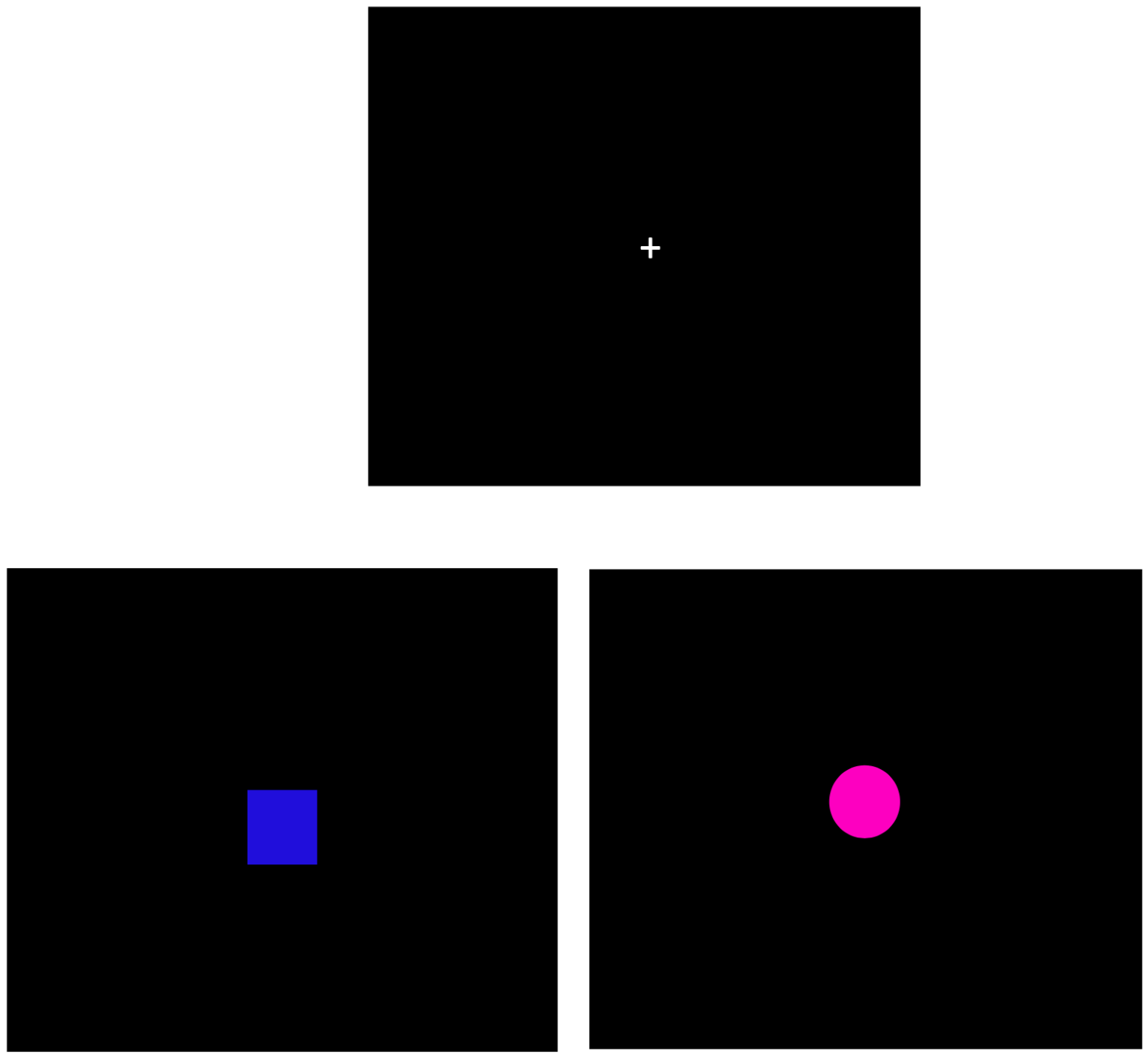

Important: When the Presentation window opens, check if the port codes are working before running the experiment. If they are not, you cannot see any events when you analyze the data. To do this, go to:

> *Settings > Port > Select Output Port 1 > Test > Send*

You should be able to see the response (1) underneath the data acquisition window on the EEG RECORDING laptop.

Once the output ports are working, return to the Main tab. For Assessment A, randomly select a task (PinkCircleGo or BlueBoxGo) and record the selection on the participant's log. For Assessment B, perform the task not performed in Assessment A. To select the task, click on the Configuration dropdown. When ready, press Run to run the experiment. You will be prompted to enter the subject ID (e.g., PA-07_PinkCircleGo_A). When ready, click OK. Now press Run Scenario for the instructions to appear full-screen on the screen. Now say:

> *[For PinkCircleGo]*
>
> “This second experiment is called a Go/No-go task, and its purpose is to assess the efficiency of response inhibition. To do so, you must focus again on the cross appearing in the middle of the screen. However, throughout this task, some figures will appear on the screen. They’re either a blue box or a pink circle. Press the mouse's left button if you see a pink circle; don’t press the button if you see a blue box. Try to do this as fast but as accurately as you can. Again, please try not to move or blink too much. You will perform a practice run before we start the recording. Please stop when you finish the

> practice and wait for us to start the recording. Whenever we start the recording, you can start with the task."

> *[For BlueBoxGo]*

> "This second experiment is called a Go/No-go task, and its purpose is to assess the efficiency of response inhibition. To do so, you must focus again on the cross appearing in the middle of the screen. However, throughout this task, some figures will appear on the screen. They're either a blue box or a pink circle. Press the mouse's left button if you see a blue box; don't press the button if you see a pink circle. Try to do this as fast but as accurately as you can. Again, please try not to move or blink too much. You will perform a practice run before we start the recording. Please stop when you finish the practice and wait for us to start the recording. Whenever we start the recording, you can start with the task."

After the practice run has ended, say:

> "You have now finished the practice run. Please wait for us to start the recording before you proceed with the task."

** Ask if they have any questions **

Important: To save the EEG acquisition, you must press Start File and then Pause Save on the EEG RECORDING laptop. A window will appear where you can choose a name for this file (e.g., PA-07_PinkCircleGo_A) and write a short description of the task (e.g., Assessment A). Also, ensure that you select the checkbox for the 8 External electrodes and the 64 Regular electrodes. It will only save the recordings when the Pause Save button is green! Once the task is over, press Pause Save again and Stop (at the top/left of the screen) so that you can start a new file for the next experiment.

As soon as you have started the recording, say:

> "We have now started the recording. Remember not to move or blink too much. Please proceed whenever you are ready."

After the EEG recording battery is done, remove the cap and invite the participant to take a 10-15 minutes break. Offer they a towel if they wish to wash or clean their hair from electrode gel. Remind them that this conductive should dissolve easily in water.

The end!

[Change to slide: Thank you!.]

Once every task is completed, remember to THANK your participant. This is very important. You're not only representing yourself as a researcher and a scientist, but you're also representing the Computational Neuroscience Research Lab. You want the participants to like you so that, if needed, you can use them again in future studies and so that they recommend

the study to their friends (this is the best way to find new participants!), but you also want them to take your tasks seriously.

ONBOARDING SCRIPT (CONTROL)
"Healthy Lifestyle Intervention"

Before starting, print the executive summary of the Physical Activity Guidelines (PAG) for Americans from the DHHS.

> "Hello, and thank you for your continued participation in our study. You have been assigned to complete the healthy lifestyle intervention using standard Physical Activity Guidelines from the U.S. Department of Health and Human Services. This session will take around 30 minutes. You will be guided through every section of this guide. The research team will ask you to read the complete executive summary of the guidelines and clarify any questions you might have."

** While holding the executive summary of the PAG, point to the first two pages. **

> "In the first two pages, you will find introductory information on the benefits of following the guidelines and their rationale."

** Point to the fourth page. **

> "On the fourth page, you will find the Key Guidelines for Adults, which you will follow. Please read them carefully and let us know if you have any questions."

** Point to the last page (page seven). **

> "On the last page, you will find a visual summary of all the information included in these guidelines.

** Close the guidelines and hand them over to the participant. **

> "Please take some time to read them and let us know if you have any questions. While you do this, we will send you a separate document to your e-mail account. This 118-page document provided by the U.S. Department of Health and Human Services contains an in-depth analysis and explanation of the guidelines provided. The printed material we are sharing with you today is the summary of this document. Feel free to read this document whenever you can and let the research team know if you have any questions."

Goals reminder

Say the following:

> "Although some of the details provided in the guideline might go beyond the goals required for this specific intervention, please remember that **in order to consider your**

intervention as complete, you are required to complete at least 80% of the days the three following goals:”

- **Stand and move** for at least 1 minute during 12 different hours daily
- **Exercise** at least 30 minutes of moderate aerobic activity daily
- **Sleep** for at least 7 hours daily

** Ask if they understood. **

Follow-up

Now proceed to schedule the weekly check-ins.

> “Before you go, we will schedule four weekly remote check-ins. These check-ins will be over a secured Zoom connection and consist of a brief interview and a questionnaire. The check-ins will help us understand your experience with the intervention at different time points and track the completion of goals. We will also ask you for any significant issues you might be facing. You will select the same time and day of the week for the four assessments. Please commit to this schedule and let us know if you face any issues in complying with it."

** Proceed to schedule a four-week recurring Zoom meeting of 15 minutes. **

Once you have finished, remember to THANK your participant. This is very important. You’re not only representing yourself as a researcher and a scientist, but you’re also representing the Computational Neuroscience Research Lab. You want the participants to like you so that, if needed, you can use them again in future studies and so that they recommend the study to their friends (this is the best way to find new participants!), but you also want them to take your tasks seriously.

ONBOARDING SCRIPT (INTERVENTION)
"Healthy Lifestyle Intervention"

Before starting, ensure the Apple Watch has been extracted from the box, and its software is updated to the latest WatchOS version (10.0.2). Also, **record the device serial number in the participant's log**.

> "Hello, and thank you for your continued participation in our study. For this portion of the study, you will be lent a smartwatch, which you will use throughout the intervention and **is to be returned when the intervention ends**. This session will take around 30 minutes and require constant use of your phone to pair the watch. You will be guided through every step of the configuration, so there is no need for the research team to touch your phone or use it without your supervision. The research team will only use your phone during onboarding under your express consent if you have technical issues or ask for assistance."

Consent for Smartwatch

Hand them the consent form and say:

> "Hello, and thank you for your continued participation in our study. For this portion of the study, you will be lent a smartwatch, which you will use throughout the intervention and is to be returned when the intervention ends. This session will take around 30 minutes and require constant use of your phone to pair the watch. You will be guided through every step of the configuration, so there is no need for the research team to touch your phone or use it without your supervision. The research team will only use your phone during onboarding under your express consent if you have technical issues or ask for assistance."

After they finish reading or signing the consent form, ask them the following questions:

> "Do you have any questions, or would you like any additional details?"
>
> *[Answer questions.]*

After signing the consent form, check that no fields are missing and hand them a copy for their records. Then, proceed to request a piece of photo ID and make a copy. Say:

> "We will now proceed to make a copy of your ID. This personal information will only be used to contact you if you do not return the smartwatch when the intervention ends. The copy will be destroyed as soon as you return the smartwatch. No other personal information will be asked for or reported on."

Make sure to return the ID. Store the consent form and copy of the photo ID in a secure location following protocol. Then, proceed with the set-up.

Fitting the device

Using the correct-sized smartwatch strap, you will help the participant secure a good fit. Say:

> "Before we begin, we will select a strap that fits your wrist size. Wearing the watch with the right fit — not too tight, not too loose, and with room for your skin to breathe — keeps you comfortable and lets the sensor on the back of the watch do their jobs more efficiently."

** Choose the appropriate wrist strap and test a good fit **

> "You might want to tighten your watch band for workouts, then loosen it a bit when you're finished. It is important that the sensors are on top of your wrist, otherwise, they won't work properly."

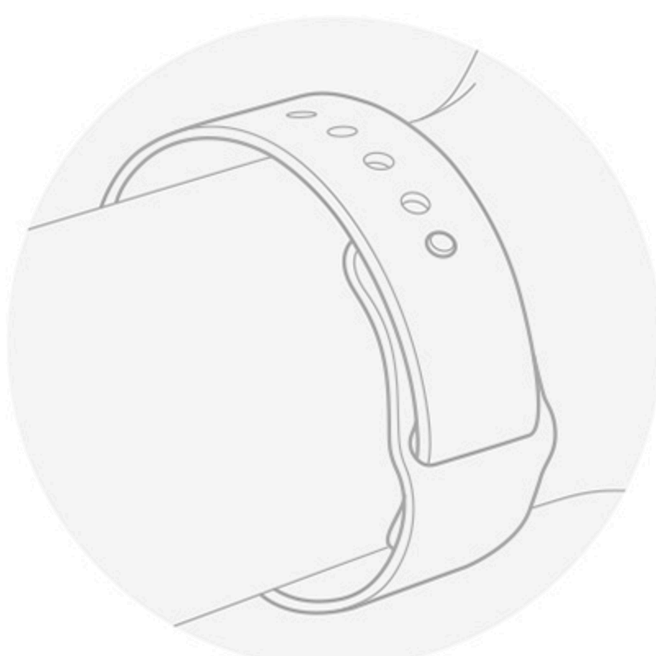

Too loose: If the watch does not stay in place or the sensors are not reading the heart rate, tighten the band. (Image source: Apple Support - https://support.apple.com/en-us/HT204665)

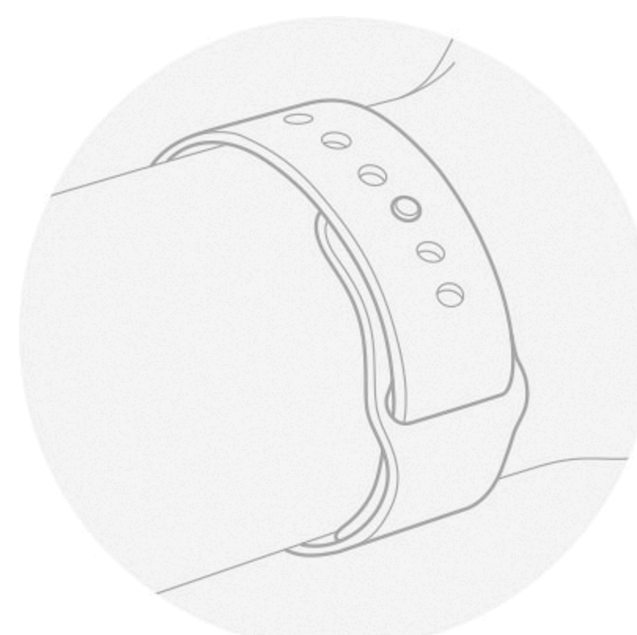

Just right: The watch should be snug but comfortable. (Image source: Apple Support- https://support.apple.com/en-us/HT204665)

Steps

After ensuring a good fit, walk through the following steps with the participant:

Pairing and goals set-up: stand, move, and exercise

1. Turn on the Apple Watch by pressing and holding the side button until the Apple logo appears.
2. Hold the Apple Watch close to the iPhone. Wait for the "Use your iPhone to set up this Apple Watch" message to appear on your iPhone, then tap Continue. If this message does not appear, open the Apple Watch app on the iPhone, tap All Watches, and then tap Add Watch.
3. Tap Set Up for Myself. Keep the Apple Watch and iPhone close until you complete all the steps.
4. Hold the iPhone over the animation. Center the watch face in the viewfinder of the iPhone. Wait for a message to say that the Apple Watch is paired.

5. Select Set Up as New Apple Watch.
6. Choose your non-dominant wrist to use the Apple Watch, and record this in the participant's log. Press Continue.
7. Read the Terms and Conditions and tap Agree.
8. Choose not to share Watch Analytics with Apple. Press Don't Share.
9. If asked, enter your Apple ID password.
10. Skip creating a passcode. Press Don't Add Passcode.
11. Customize your settings. Select your preferred reading experience and press Continue.
12. **The following steps will vary as they depend on the current settings on the iPhone.** Please select all that apply to you and the study. **Stop on the Personalize Fitness and Health.**
13. Add or update details of date of birth, sex, height, and weight to ensure that the Fitness and Health data are accurate. Press continue.
14. In the Activity step, press Set Up Activity.
15. Set up your Daily Move Goal. This goal is based on the personal details provided and any data you might have stored on your iPhone. We suggest selecting the one provided as a default. **Record this selection on the participant log.** Press Set Move Goal.
16. Set up a **Daily Exercise Goal of 30 minutes**. Press Set Exercise Goal.
17. Set up a **Daily Stand Goal of 12 hours/day**. Press Set Stand Goal.
18. Allow Workout Route Tracking. Press Enable Route Tracking.
19. Turn on all Health Notifications (Noise, Low Heart Rate, and High Heart rate). Press Continue.
20. Read information about Safety and Always On Display. Press Continue.
21. Select the default Grid View App View. Press Continue.
22. If prompted to set up Apple Pay, press Set Up Later.
23. Read the information on the Welcome to Apple Watch screen. Press OK.

Activate activity notifications

1. You will now be redirected to the Watch app My Watch tab. Select Notifications.
2. Go to Activity. Be sure that Allow Notifications is selected. Turn on all notifications (Stand Reminders, Daily Coaching, Goal Completions, Special Challenges, and Activity Sharing Notifications).

Sleep configuration and goal setup

1. Please return to the main Watch app, My Watch tab. Select Sleep. Make sure that Track Sleep and Charging Reminders are turned on.
2. Exit the Watch app and go into the Health App (Install the app if not installed).
3. Tap the Browse tab in the bottom-right corner of the screen.
4. Scroll down and select Sleep.
5. Under "Your Schedule," tap Full Schedule & Options. If this is the first time using the feature, tap "Get Started."
6. Make sure that the Sleep Schedule is turned on. Under Additional Details, tap Sleep Goal and **select a goal of 7 hours**.

Share health data with the Research Team

1. Ask the participant to add “ail7@sfu.ca” to their contacts. This account will be solely used to monitor health data during the intervention and will be deleted afterward.
2. On the Health app, tap the Sharing tab, then tap Share with Someone. Make sure that Health is turned on in the iCloud account.
3. Search for the contact “ail7@sfu.ca”. It should appear in blue. Select it.
4. Tap Set Up Manually.
5. In Health Alerts, make sure that no option is selected. Press Next.
6. Under Activity, turn on Activity, Exercise Minutes, Stand Hours, Stand Minutes, and Steps.
7. Under Sleep, turn on Sleep. Now scroll down and press Next.
8. Before the participant starts sharing data, invite them to press Preview. That way, they will know what type of data will be shared with us during the study. This preview will always be visible during the intervention, and we cannot turn on more data remotely. However, remind them to leave all the requested data sharing.
9. When they are ready, press Share.

Recommendations

- They should not shower or swim while using the watch to avoid water intake. It is okay if the watch gets wet from sweat, rain, or water splashes.
- We recommend charging the watch while taking a shower or sitting at a desk. You need to use the watch while sleeping, so securing a charge that lasts until you are awake is essential.
- Do not update the WatchOS during the intervention.
- Remind them they can personalize the watch to fit their goals during the intervention but not change any initial set-up or goals.
- Keep a registry of any decision or personalization you made while using the watch. We will ask questions about this at every weekly check-in.
- Remember not to unshare any of the selected data during the intervention.

Goals reminder

Remind them that the goals they are expected to complete are the following:

- **Stand and move** for at least 1 minute during 12 different hours daily
- **Exercise** at least 30 minutes of moderate aerobic activity daily
- **Sleep** for at least 7 hours daily

They should achieve each goal at least 80% of the days for the intervention to be considered complete.

** Ask if they understood. **

Follow-up

Now proceed to schedule the weekly check-ins.

> “Before you go, we will schedule four weekly remote check-ins. These check-ins will be over a secured Zoom connection and consist of a brief interview and a questionnaire. The check-ins will help us understand your experience with the intervention at different time points and track the completion of goals. We will also ask you for any significant issues you might be facing. You will select the same time and day of the week for the four assessments. Please commit to this schedule and let us know if you face any issues in complying with it."

** Proceed to schedule a four-week recurring Zoom meeting of 15 minutes. **

Once you have finished, remember to THANK your participant. This is very important. You’re not only representing yourself as a researcher and a scientist, but you’re also representing the Computational Neuroscience Research Lab. You want the participants to like you so that, if needed, you can use them again in future studies and so that they recommend the study to their friends (this is the best way to find new participants!), but you also want them to take your tasks seriously.

CHECK-IN CALL SCRIPT

"Healthy Lifestyle Intervention"

Prepare the Check-In Form and pre-fill the participant's ID and Current Week fields.

Link to Check-In Form: https://forms.gle/oaQuNrXgzBztgQ1P7

Semi-structured interview (10 min)

When the participant enters the Zoom call, greet the participant and thank them for their time.

> "I will ask you questions about your experience during this week. After that, I will ask you to answer a brief survey. This Zoom call will be recorded to avoid missing what you say. Only the audio recording will be kept. You can opt out of the audio recording if you are unwilling to be recorded. In that case, I will turn off the recording when you talk and take notes instead. Please remember that recordings will be saved using a participant code instead of your name. A master list that links the code to your name will be kept during the study and destroyed whenever the results are published. No other record linking your name to your participant code will be maintained after the study is published."

** Ask if they understood **

> "Do you consent to be audio recorded during this interview?"

If they are okay with recording, start recording now. If not, proceed with the interview and try taking detailed notes of the interview.

Now, follow as best as possible the next questions in a natural and conversational pace. Ensure that you get answers for all or most of the questions whenever possible:

1. Did you face any issues following the intervention this week?
    a. What did you do about them?
2. Was there something particularly useful that helped you follow the intervention this week?
    a. How did you discover this?
    b. Did you change or customize any aspect of the intervention?

Goal completion survey (5 min)

Now, proceed to ask and capture the answers to the six questions in the Check-in Form.

> "Now, I will ask six brief survey questions to measure your progress toward completing the intervention."

** Complete the survey. After this, ask them if they have any questions **

** Stop the recording. Tell the participant that you have stopped recording **

Once the check-in is completed, remember to THANK your participant. This is very important. You're not only representing yourself as a researcher and a scientist, but you're also representing the Computational Neuroscience Research Lab. You want the participants to like you so that, if needed, you can use them again in future studies and so that they recommend the study to their friends (this is the best way to find new participants!), but you also want them to take your tasks seriously.

STUDY SCRIPT (POST)
"Healthy Lifestyle Intervention"

[Before the participant arrives, show slide with the study title.]

Greet the participant and welcome them to the lab. Remind them that if they want to go to the bathroom they should go now, as afterward it might be complicated because of the required set-up. Ask them to sit down in front of the stimulus computer and align the chair to the marks on the floor. Check that the center of the computer screen is leveled to their eyes, otherwise make necessary adjustments. Request them to turn off their phones or any device or notification that might distract them during the assessment. Ask them if they are comfortable, then proceed. Have a timer at hand to record the length of each procedure.

SF-36 quality of life (Do this while capping)

[Change to slide: EEG Capping.]

While capping, try to explain some of the steps to the subject. Keep them engaged and not bored. It is not good if they're bored before the tasks start. Say:

> "We want to pick up some of the electricity that your brain gives off. To do so, we will put this cap on your head. It will feel just like a swimming cap. We will start by placing two electrodes on your temples (point on your face), one beneath your left eye (point on your face), and two on your mastoids (point again on your neck). We do this to measure electrical activity given by the muscular movements around your eyes and neck. We will have to clean the area to get a better signal. We will also do some measurements of your head to select a right-sized cap."

Do it

Please remember to:

- Clean the temples and mastoids with an alcohol swipe (or an existing alternative);
- Measure the circumference of the head (the measuring tape must go over the inion and the nasion). This will allow you to choose the right-sized cap (if someone is on the limit, always choose the smallest of both options). Make a record of the selected size;
- Glue circular stickers onto external electrodes (1-5) with the center of the sticker coinciding with the center of the electrode and add some gel to the area;
- Put external electrodes 1 on the left temple, 2 on the right temple, 3 below the left eye (1.5 cm below the eyelid), 4 on the right mastoid, and 5 on the left mastoid. Remember to try to put these on with the wires directed to the back of the head so that they can be tucked behind the subject's ear;
- Put the cap on the subject's head. Ask them to help by holding the Fp1, Fpz, and Fp2 electrodes against her forehead. (Directions below)

"Now, we'll put the cap on you, and we will put some gel into these holes (show the holes) because the electrodes need a medium to conduct the electrical signal from the scalp to the metal (show them how). I'll need your help. I'll need you to hold this front part against your forehead, please. (Place the three frontal electrodes of the cap in the correct place and let them hold it down while you pull the back part of the cap down – be sure that the cap is well applied by verifying the midline both longitudinally and transversally)."

[Change to slide: Health Survey.]

Show the volunteers the SF-36 questionnaire and say:

> "While we finish setting up, we would like to understand better how you feel, how well you can do your usual activities, and how you rate your own health. To help us better understand these things about you, we will ask you some questions about your general health. The survey will be done on the same laptop. Please click on 'Click here to begin test.' (Wait for the survey to open) This is not a test, and there are no right or wrong answers. Choose the response that best represents the way you feel. Please answer every question. As we proceed, please feel free to ask me any questions you may have."

While continuing to cap, please remember to:

- Start by gelling all the labeled electrodes.
- Insert the electrodes in the cap (two sets of 32 electrodes (A and B)). Be careful so that none of the electrodes touch metallic surfaces, and remember that they are very fragile (that's why we call them "angel hair"). It might be easier to put the electrodes around your neck so that there's not a lot of weight on them.
- Finally, insert DRL and CMS electrodes.
- Test the EEG recording signal.

After having a good signal from every electrode, proceed to the next task.

System Usability Scale (Do this while capping)

[Change to slide: System Usability Scale.]

As soon as the participant is finished with the previous task, show the volunteers the System Usability Scale and say:

> "While we continue setting up, we would like you to answer a short survey that will help us better understand your experience during the intervention. Please answer all the questions honestly and without interruptions. The survey will be done on the same laptop. Please click on 'Click here to begin test.' (Wait for the survey to open) As we proceed, please feel free to ask any questions you may have."

RestingState (5min)

[Change to slide: Resting State Recording.]

Say:

> "We are going to start with a simple task. This task aims to measure electrical activity in your brain while resting. Once it begins, a cross will appear in the center of the screen. You just have to focus on it for one minute. Please try not to move or blink too much. You can reread the instructions when we set up the recording."

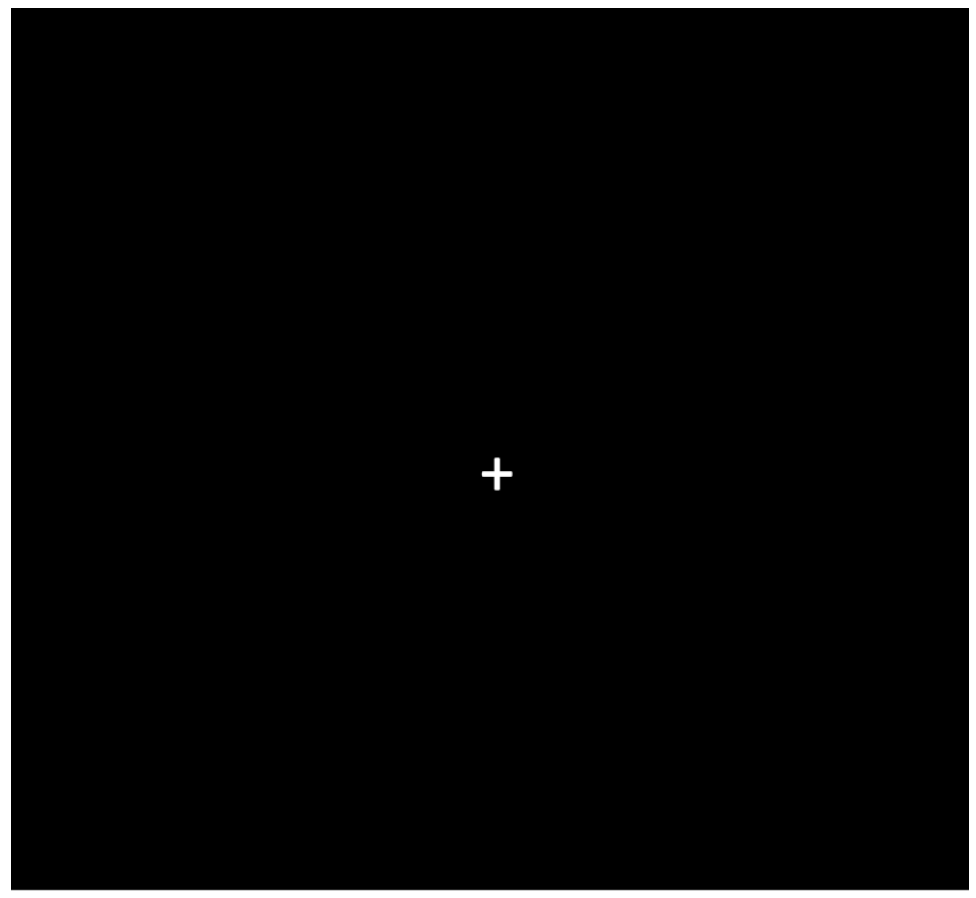

Open the Presentation files on the PRESENTATION laptop to open the Resting State task.

> *CNRL_SW_Presentation > Resting State > Resting State.sce*

Important: When the Presentation window opens, check if the port codes are working before running the experiment. If they are not, you cannot see any events when you analyze the data. To do this, go to:

> *Settings > Port > Select Output Port 1 > Test > Send*

You should be able to see the response (1) underneath the data acquisition window on the EEG RECORDING laptop.

Important: To save the EEG acquisition, you must press Start File and then Pause Save on the EEG RECORDING laptop. A window will appear where you can choose a name for this file (e.g., PA-07_RestingState_A) and write a short description of the task (e.g., Assessment A). Also, ensure that you select the checkbox for the 8 External electrodes and the 64 Regular electrodes. It will only save the recordings when the Pause Save button is green! Once the task is over, press Pause Save again and Stop (at the top/left of the screen) so that you can start a new file for the next experiment.

Once the output ports are working and you have started the recording, return to the Main tab and press Run to run the experiment. Then click Run Scenario for the instructions to appear full-screen on the screen. Now say:

> "After you complete each task, a new set of instructions will appear. Please read them carefully before proceeding. When you are ready, please click anywhere on the screen to begin."

When the minute has passed and the instructions for the second task appear on the screen, say:

> "We have finished the first task. Now, we will do another one-minute recording, but this time, we need you to close your eyes during the recording. Try not to move your eyes or your head during the recording. We will let you know when you can open your eyes again. When you are ready, please click anywhere on the screen to begin."

When the minute has passed, indicate the participant to open their eyes and thank them for following indications. Now, proceed to stop the recording on the EEG RECORDING laptop and finish the experiment on the PRESENTATION laptop.

Go/No-Go (10 min)

[Change to slide: Go/No-Go Task.]

Open the Presentation files on the PRESENTATION laptop to open the Go/No-Go task.

> *CNRL_SW_Presentation > Go-NoGo > Go-NoGo Task > Go-NoGo Task.exp*

Important: When the Presentation window opens, check if the port codes are working before running the experiment. If they are not, you cannot see any events when you analyze the data. To do this, go to:

> *Settings > Port > Select Output Port 1 > Test > Send*

You should be able to see the response (1) underneath the data acquisition window on the EEG RECORDING laptop.

Once the output ports are working, return to the Main tab. For Assessment A, randomly select a task (PinkCircleGo or BlueBoxGo) and record the selection on the participant's log. For Assessment B, perform the task not performed in Assessment A. To select the task, click on the Configuration dropdown. When ready, press Run to run the experiment. You will be prompted to enter the subject ID (e.g., PA-07_PinkCircleGo_A). When ready, click OK. Now press Run Scenario for the instructions to appear full-screen on the screen. Now say:

> *[For PinkCircleGo]*
>
> "This second experiment is called a Go/No-go task, and its purpose is to assess the efficiency of response inhibition. To do so, you must focus again on the cross appearing in the middle of the screen. However, throughout this task, some figures will appear on the screen. They're either a blue box or a pink circle. Press the mouse's left button if you see a pink circle; don't press the button if you see a blue box. Try to do this as fast but

as accurately as you can. Again, please try not to move or blink too much. You will perform a practice run before we start the recording. Please stop when you finish the practice and wait for us to start the recording. Whenever we start the recording, you can start with the task."

[For BlueBoxGo]

"This second experiment is called a Go/No-go task, and its purpose is to assess the efficiency of response inhibition. To do so, you must focus again on the cross appearing in the middle of the screen. However, throughout this task, some figures will appear on the screen. They're either a blue box or a pink circle. Press the mouse's left button if you see a blue box; don't press the button if you see a pink circle. Try to do this as fast but as accurately as you can. Again, please try not to move or blink too much. You will perform a practice run before we start the recording. Please stop when you finish the practice and wait for us to start the recording. Whenever we start the recording, you can start with the task."

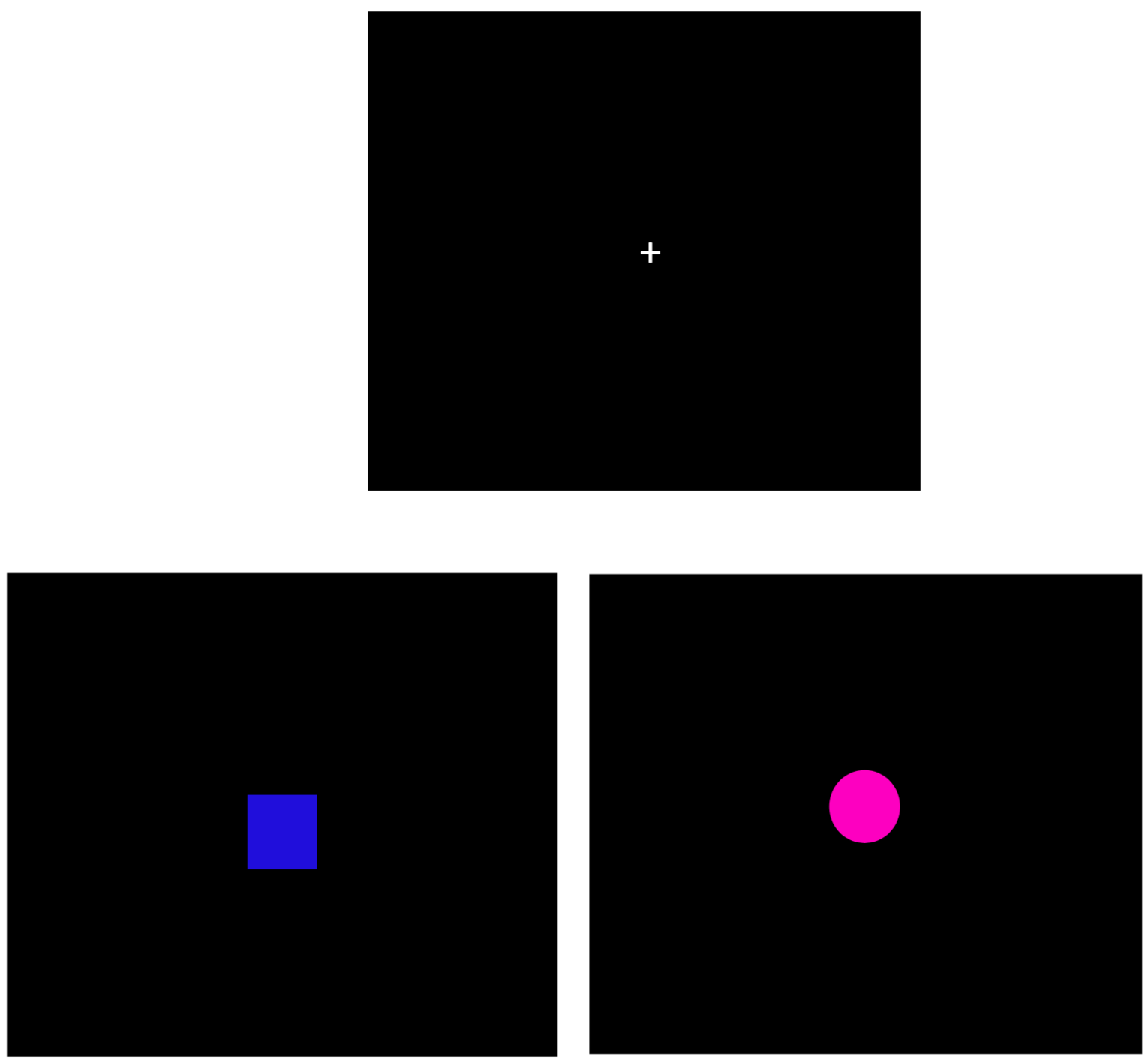

After the practice run has ended, say:

"You have now finished the practice run. Please wait for us to start the recording before you proceed with the task."

** Ask if they have any questions **

Important: To save the EEG acquisition, you must press Start File and then Pause Save on the EEG RECORDING laptop. A window will appear where you can choose a name for this file

(e.g., PA-07_PinkCircleGo_A) and write a short description of the task (e.g., Assessment A). Also, ensure that you select the checkbox for the 8 External electrodes and the 64 Regular electrodes. It will only save the recordings when the Pause Save button is green! Once the task is over, press Pause Save again and Stop (at the top/left of the screen) so that you can start a new file for the next experiment.

As soon as you have started the recording, say:

> “We have now started the recording. Remember not to move or blink too much. Please proceed whenever you are ready.”

After the EEG recording battery is done, remove the cap and invite the participant to take a 10-15 minutes break. Offer they a towel if they wish to wash or clean their hair from electrode gel. Remind them that this conductive should dissolve easily in water.

Semi-structured interview (30-60min)

[Change to slide: Interview.]

When the break is done, ask them to sit down in the room designated for the interview and say:

> "I will ask you questions about your experience, preferences, and feelings after completing the intervention. I will take handwritten notes to record your answers and use an audio recorder to ensure we don’t miss what you say. You can opt out of the audio recording if you are not willing to be recorded. In that case, I will turn off the field recorder when you talk and take notes instead. Please remember that recordings will be saved using a participant code instead of your name. A master list that links the code to your name will be kept during the study and destroyed whenever the results are published. No other record will be maintained linking your name to your participant code after the study is published."

Ask if they understood

> “Do you consent to be audio recorded during this interview?”

If they are ok with recording, start recording now. If not, proceed with the interview and try taking detailed notes of the interview.

Now, follow as best as possible the next questions in a natural and conversational pace. Ensure that you get answers for all or most of the questions whenever possible:

1. Before we begin, could you tell me a bit about yourself?
    a. Do you live alone or with anyone else?
    b. What do you do for a living?
2. Please walk me through what a typical day looks like.
    a. How do you decide on your activities?
    b. Are you typically alone or with others?

   c. How do you choose to rest/stay active?
3. You mentioned you [had/hadn't] difficulties following the intervention. Why is that?
   a. Tell me more about that.
   b. Was that important to you?
   c. Why?
4. For your everyday schedule, how do you decide to stay active?
   a. How do you decide your sleeping habits?
   b. Do your plans ever change?
   c. Can you give me an example of a time when they changed?
   d. Why did you need to change your plans?
   e. How did you feel about that?
5. Was there a particular time when following the intervention was hard?
   a. When was this?
   b. What did you do instead?
   c. How did you feel about that?
6. How frequently did you stop your regular activities to follow the intervention?
   a. How did you feel about it?
7. Overall, would you say that following the intervention improved your overall health?
   a. Why?
   b. Would you say it increased your physical activity?
   c. How?
   d. Was there any particular feature from the watch/guidelines that helped you achieve this?
   e. Why?
   f. Did it improve your quality of sleep?
   g. How?
   h. Was there any particular feature in the watch/guidelines that helped you change this?
   i. Why?
8. If there's one thing you could change from the intervention, what would that be?
   a. How would that make it easier for you?
   b. Tell me more about that.
   c. Why is that important to you?
9. Do you have anything else you'd like to share about this topic?
   a. What will happen now that you no longer have access to the intervention?
   b. Would you consider continuing to follow the intervention?

You have now finished the interview. Stop the recording and tell the participant that you have now stopped recording. While you do this, consider if a 5-minute break is necessary before proceeding with any further steps.

Smartwatch Set-Away (Only for intervention group)

In this stage, you will ask the participant to stop using the smartwatch and return any accessory the research team might have lent them, including charger, straps, and boxes. You will then proceed to back-up the data from the watch, stop the sharing of real-time activity data from the participant's phone with the research team, and then unpair the watch from the participant's phone.

> "Now we will proceed to back-up the health data gathered by the smartwatch. We need this data to analyze your activities and completion of goals during the intervention. This will help us understand how effective the intervention was when facilitated by a smartwatch with personalized healthy lifestyle prompts. This step will take around 15 minutes and require constant use of your phone to back-up the data from the watch, stop the sharing of real-time activity data from your phone with the research team, and then unpair the watch from your phone. You will be guided through every step of the configuration, so there is no need for the research team to touch your phone or use it without your supervision. The research team will only use your phone during this step under your express consent if you have technical issues or ask for assistance. Remember that after analysis, all health and fitness data will be de-identified, and no published data will link back to participants' identities. Any Apple servers' backups and services will be deleted and terminated after the study is published."

** Ask them if they have any questions **

<u>Step 1: Back-up health and fitness data</u>

1. On your iPhone, open the Health app.
2. On the top right corner, tap your iCloud profile picture.
3. Scroll down to the bottom. Press Export All Health Data. You will be asked to confirm this action, press Export. It will take around 30 seconds for the data to be exported.
4. When the export is finished, a sharing modal will pop from the bottom. Select the preferred sharing method. We recommend sharing this export through Airdrop to the research lab's computer.
5. After the transfer is done, check that the exported file can be uncompressed and that all files are complete and in a good state.
6. You can now go back to the Health's app initial screen.

<u>Step 2: Stop sharing health and fitness data</u>

1. In the Health's app bottom navigation bar, select Sharing.
2. Under the "You're Sharing With" section, select the CNRL account.
3. A screen with all the data you are sharing will pop-up. Scroll all the way to the bottom. Select Stop Sharing. You will be asked to confirm this action, select Stop Sharing. This will delete all the Health data from the CNRL account.

<u>Step 3: Unpair and erase the Apple Watch</u>

1. If you have any transit card or banking card in the Wallet app, remove them from the Apple Watch before you unpair. This is only a safety measure recommended by Apple. Remember that the research team never had access to this, or any other sensitive information other than the health and fitness data, user-entered data, and general system settings visible at any time in the shared data preview.
    a. To remove a card, open the Wallet app on the Apple Watch. Tap the card that you want to remove, then scroll down and tap Remove.

2. Keep the Apple Watch and iPhone close together as we unpair them.
3. Open the Apple Watch app on the phone.
4. Go to the My Watch tab and tap All Watches.
5. Tap the info button (i) next to the watch that you want to unpair.
6. Tap Unpair Apple Watch.
7. Tap Unpair [Apple Watch name].
8. Type your Apple ID password to disable Activation Lock, then tap Unpair.
9. Before erasing all the content and settings on the Apple Watch, the iPhone creates a backup of the Apple Watch. You will be able to use this backup to restore a new Apple Watch if you decide to get one.
10. Turn off the Apple Watch. Press and hold the side button until the sliders appear. Tap the power symbol, then drag the Power Off slider to the right.
11. Store the watch and any accessories in a secure location. Be sure to disinfect it with a 70 percent isopropyl alcohol wipe, and clean with a nonabrasive, lint-free cloth.

The end!

[Change to slide: Thank you!.]

Once every task is completed, remember to THANK your participant. This is very important. You're not only representing yourself as a researcher and a scientist, but you're also representing the Computational Neuroscience Research Lab. You want the participants to like you so that, if needed, you can use them again in future studies and so that they recommend the study to their friends (this is the best way to find new participants!), but you also want them to take your tasks seriously.
