# Supplementary material for: Assessing the Effectiveness, Engagement, and Acceptability of a Smartwatch Healthy Lifestyle Intervention for Cognitive Enhancement in Healthy Adults: Mixed Methods Electroencephalography Protocol
Source: JMIR Res Protoc. 2026 Nov 6;15:e81490. doi: 10.2196/81490 (PMC13753865; doi:10.2196/81490)
Supplement: Supplementary file 2 [file resprot-v15-e81490-s002.pdf]

36-Item Short Form Survey Instrument (SF-36)

RAND 36-Item Health Survey 1.0 Questionnaire Items

Choose one option for each questionnaire item.

1. In general, would you say your health is:

- 1 - Excellent
- 2 - Very good
- 3 - Good
- 4 - Fair
- 5 - Poor

---

2. **Compared to one year ago**, how would you rate your health in general **now**?

- 1 - Much better now than one year ago
- 2 - Somewhat better now than one year ago
- 3 - About the same
- 4 - Somewhat worse now than one year ago
- 5 - Much worse now than one year ago
